# Methodological approaches to study context in intervention implementation studies: an evidence gap map

**DOI:** 10.1186/s12874-022-01772-w

**Published:** 2022-12-14

**Authors:** Juliane Mielke, Thekla Brunkert, Franziska Zúñiga, Michael Simon, Leah L. Zullig, Sabina De Geest

**Affiliations:** 1 Institute of Nursing Science, Department Public Health, University of Basel, Bernoullistrasse 28, CH-4056 Basel, Switzerland; 2 University Department of Geriatric Medicine FELIX PLATTER, Basel, Switzerland; 3 Center for Innovation to Accelerate Discovery and Practice Transformation (ADAPT), Durham Veterans Affairs Health Care System and Department of Population Health Sciences, Duke University Medical Center, Durham, NC USA; 4 Department of Public Health and Primary Care, KU Leuven, Academic Center for Nursing and Midwifery, Louvain, Belgium

**Keywords:** Implementation science, Contextual analysis, Dissemination, Evidence gap map

## Abstract

**Background:**

Within implementation science studies, contextual analysis is increasingly recognized as foundational to interventions' successful and sustainable implementation. However, inconsistencies between methodological approaches currently limit progress in studying context and guidance to standardize the use of those approaches is scant. Therefore, this study's objective was to systematically review and map current methodological approaches to contextual analysis in intervention implementation studies. The results would help us both to systematize the process of contextual analysis and identify gaps in the current evidence.

**Methods:**

We conducted an evidence gap map (EGM) based on literature data via a stepwise approach. First, using an empirically developed search string, we randomly sampled 20% of all intervention implementation studies available from PubMed per year (2015–2020). Second, we assessed included studies that conducted a contextual analysis. Data extraction and evaluation followed the Basel Approach for CoNtextual ANAlysis (BANANA), using a color-coded rating scheme. Also based on BANANA and on the Context and Implementation of Complex Interventions (CICI) framework–an implementation framework that pays ample attention to context– we created visual maps of various approaches to contextual analysis.

**Results:**

Of 15, 286 identified intervention implementation studies and study protocols, 3017 were screened for inclusion. Of those, 110 warranted close examination, revealing 22% that reported on contextual analysis.

Only one study explicitly applied a framework for contextual analysis. Data were most commonly collected via surveys (*n* = 15) and individual interviews (*n* = 13). Ten studies reported mixed-methods analyses. Twenty-two assessed meso-level contextual and setting factors, with socio-cultural aspects most commonly studied. Eighteen described the use of contextual information for subsequent project phases (e.g., intervention development/adaption, selecting implementation strategies). Nine reported contextual factors' influences on implementation and/or effectiveness outcomes.

**Conclusions:**

This study describes current approaches to contextual analysis in implementation science and provides a novel framework for evaluating and mapping it. By synthesizing our findings graphically in figures, we provide an initial evidence base framework that can incorporate new findings as necessary. We strongly recommend further development of methodological approaches both to conduct contextual analysis and to systematize the reporting of it. These actions will increase the quality and consistency of implementation science research.

**Supplementary Information:**

The online version contains supplementary material available at 10.1186/s12874-022-01772-w.

## Background

Successful implementation of interventions in real-world settings depends on the dynamic, multi-dimensional, multi-level interplay between context, intervention and implementation strategies [1, 2]. Therefore, a thorough understanding of the implementation context is critical. This is true not only for the initial implementation, but also for sustainability and scale-up [3–7]. Filling this need is the role of contextual analysis, i.e., the mapping of multi-dimensional and multi-level contextual factors relevant for the implementation of an intervention in a specific setting.

Within an implementation science^1^ project, we understand contextual analysis as a separate study. It starts well before implementation and continues throughout the project. The in-depth contextual knowledge informs subsequent phases of the project, especially the development or adaption of an intervention and choices of implementation strategies [8–10]. Within that setting, contextual analysis helps to interpret the studied intervention's effectiveness and implementation outcomes and guides choices of sustainability strategies [11, 12].

Although the importance of context has been widely emphasized regarding implementation, little attention has been paid to its assessment in studies partly driven by funding frameworks that do not normally recognize this phase's importance [13–15]. Yet, conceptual and methodological challenges hamper the assessment of context additionally. Even the concept of context is only partially mature [16–18]: a recent systematic review revealed inconsistencies in current theoretical and operational definitions [18].

No unifying definition of context yet exists. Instead, we see terms including setting—sometimes divided into inner and outer setting—environment, or system characteristics, with each signifying a slightly different perspective [16, 19, 20]. Further, no explicit methodological guidance yet describes how to assess, analyze or report context and setting.

Within a postpositivist paradigm, researchers tend to focus on single factors (commonly referred to as facilitators and barriers) to the exclusion of those occupying multiple levels and dimensions [18, 20, 21]. These factors are often selected without theoretical support; and even where contextual analyses are conducted, the findings are rarely used to inform subsequent project phases (e.g., implementation strategy choices). Additionally, no specific methods to study contexts are described, the range of psychometrically sound measurement tools (particularly to assess macro-level factors) limited, and reporting guidelines (e.g., Standards for Reporting implementation Studies (StaRI) [22, 23]) ambiguous regarding how contextual analysis to report [18, 24].

Based on a methodology reported by Stange and Glasgow [5] within a series of patient-centered medical home research for the US Agency for Healthcare Research and Quality (AHRQ), we developed the Basel Approach for CoNtextual ANAlysis (BANANA) and applied it successfully in two implementation science projects [25–27]. BANANA provides methodological guidance for contextual analyses and can point to relevant aspects in reporting contextual analyses. This approach's theoretical grounding is the Context and Implementation of Complex Interventions (CICI) framework [2], a meta-framework incorporating insights from previous frameworks (e.g., the Consolidated Framework for Implementation Research [19]), but also filling previous gaps (e.g., differentiating between context and setting, focusing more on macro-level factors, considering how other interventions can affect implementation). Starting from an ecological perspective, the authors conceptualized context as a “set of characteristics and circumstances that consist of active and unique factors, within which the implementation is embedded” [2], whereas setting refers to the physical location in which an intervention is to be implemented and interacts with both context and implementation [2]. Context “is an overarching concept, comprising not only a physical location but also roles, interactions and relationships at multiple levels” [2]. Contextual factors can be grouped into geographical, epidemiological, socio-cultural, socio-economic, political, legal or ethical domains, and include, e.g., the social structure, financial aspects, or the political climate.

To guide contextual analysis in implementation science projects, BANANA includes six components: (1) choosing a theory, model or framework (TMF) to guide contextual analysis. (To enhance analytical granularity, the TMF can be complemented with one that is setting-specific.); (2) reviewing empirical evidence about relevant contextual factor(s), including facilitators and barriers, as well as practice patterns related to the implementation and intervention; (3) involving relevant stakeholders in the contextual analysis. This includes implementation agents, i.e., individuals (or organizations) targeted or affected by the implementation of an intervention (*target group*, e.g., patients, family caregivers), who implement an intervention (*implementers*, e.g., healthcare professionals) or who decide on the implementation of an intervention (*decision makers*, e.g., policy makers and funders) [2]. Other stakeholders can include experts with advisory roles within the project (e.g., for intervention development); (4) collecting and analyzing data, combining qualitative and quantitative methods where appropriate; (5) identifying and describing the relevance of contextual factors for intervention co-design, implementation strategies and outcomes; and (6) reporting the contextual analysis [27]. To strengthen the methodology for contextual analysis in implementation science, we recognized that it would be essential first to understand the key methods currently in use. Therefore, we set out to gather an evidence base. To identify gaps in that base, we systematically reviewed and mapped the methodological approaches described. More specifically, first, we aimed to determine the percentage of published intervention implementation studies reporting on contextual analysis. Second, we aimed to assess, map and evaluate those studies that reported on contextual analysis. We focused on a) which methodological approaches were used for contextual analyses and what gaps exist in current approaches, and b) which results were used to inform subsequent phases of the associated studies.

## Methods

To draft an evidence gap map (EGM) we reviewed and categorized the methodologies applied to contextual analyses in the identified studies. This process was basically a systematic search that included surveying the current state of methodological approaches to contextual analysis. As the name implies, this was very useful to identify gaps in those approaches [28–30]. As for the mapping aspect, the results are presented in a user-friendly format, usually combining tables or visualizations and descriptive reports to summarize existing evidence and facilitate methodological improvements regarding the topic—in this case, contextual analysis [28–31]. We reported our findings according to the Preferred Reporting Items for Systematic reviews and Meta-Analyses–Scoping Reviews (PRISMA–ScR) Checklist (Additional file 1) [32].

### Scope of the evidence gap map (EGM) and development of research questions

As a first step, to develop comprehensive, relevant research questions, this study's authors—all experienced implementation science scientists—discussed the scope and focus of the EGM [31, 33]. As noted, a stepwise approach helped us identify relevant literature and provide a comprehensive overview of the available evidence (Additional file 2): First, we aimed to identify intervention implementation studies and assessed whether they included contextual analyses (Step 1). Second, focusing exclusively on studies that reported contextual analyses, we mapped both the researchers' methods (Step 2a) and how they used the results to inform further phases of their projects (Step 2b).

### Inclusion/ exclusion criteria

In step 1, we employed ten inclusion criteria to the prospective sample. We included (a) peer-reviewed articles or study protocols (b) concerning intervention implementation studies (c) if they employed experimental or quasi-experimental designs (d) to test intervention effectiveness (e) in real world settings. They also needed (f) to include at least one of Brown et al.'s "7 Ps" [34], i.e., programs, practices, principles, procedures, products, pills, and policies, and (g) to report on the evaluation of the implementation pathway. This included qualitative or quantitative information on the implementation process and/or on at least one implementation outcome as defined by Proctor et al. [35] (Additional file 2). During the screening we identified a large number of feasibility studies that did not fit the scope of our study. Therefore, we decided only to include feasibility studies (h) if they assessed at least one additional implementation outcome (e.g., feasibility *and* acceptability). Further, only papers (i) written in English or German and (j) with available full texts were included. Because the level of detail of contextual analysis in study protocols is usually limited, we used the "cited by" function in PubMed to determine whether the intervention study had been already published and contained further information on contextual analysis. In cases where we identified the study protocol and related intervention implementation study, only the intervention study was included in the review. Further, we excluded studies reporting on context exclusively as part of the process evaluation or retrospectively.

### Systematic searching – search strategy development

We applied Hausner et al.'s empirical-based approach [36] to develop our search strategy. Following a four-step process, we first used a precise search string to identify a subset of 163 articles in Pubmed that met our EGM's inclusion criteria (Additional file 3). Those articles were randomly assigned to a development (*n* = 81) or a validation set (*n* = 82). Second, using Pubmed ReMiner (https://hgserver2.amc.nl/cgi-bin/miner/miner2.cgi), we identified the search terms (keywords and MeSH terms) most commonly used in the development set articles. The identified search terms were used to develop a search string. In a third step, this string was tested against the validation set. The final search string consisted of 22 keywords (MeSH and free terms) and achieved a sensitivity of 95.1% (i.e., it identified 75 of the 81 development records). The fourth step consisted of documenting the search string development (Additional file 3).

Our main aim was to identify and map gaps in the current evidence base on approaches to contextual analysis and not to provide an exhaustive overview on all existing evidence. Therefore, we searched only the PubMed electronic database. Further, to maximize timeliness, we limited our search to the past six years (2015–2020). Using a random number generator, we then selected a random sample of 20% of the articles identified from each year. No further filters were applied.

### Study selection

For step 1, using the web application Rayyan (https://rayyan.qcri.org), two reviewers (JM, TB) independently screened titles and abstracts of the randomly selected implementation science papers against the described inclusion criteria [37]. Second, each reviewer (JM, TB) independently screened the full texts of all included papers. In case of disagreement between the two reviewers, a third reviewer (SDG) was consulted to reach consensus. For step 2, the first two reviewers (JM, TB) independently screened the full texts of previously included intervention implementation studies against the respective eligibility criteria. Again, the third reviewer (SDG) was consulted in case of disagreement.

### Data extraction and analysis

We extracted the general data of all included intervention implementation studies (e.g., design, setting). Guided by BANANA, specific characteristics of studies including contextual analyses were extracted, including general information (e.g., whether context was analyzed at various timepoints, TMFs used), implementation agents involved in each analysis and methods applied to conduct contextual analysis (i.e., quantitative and qualitative methods). Further, we assessed the results of the contextual analyses, i.e., we noted how those results were used for subsequent study phases and whether the researchers had considered how contextual factors might influence implementation and summative outcomes (Additional file 2). As it quickly became clear that few studies explicitly reported the use of hybrid designs, we used Curran et al.'s description to categorize these in the remainder that we checked, i.e., as hybrid type 1/2/3^2^ [38]. All extracted data were charted in an Excel file. General study characteristics were analyzed descriptively, calculating frequencies and percentages.

### Mapping of identified methodological approaches

We mapped the identified approaches to contextual analyses against the components of BANANA. To provide a user-friendly format, we created color coded tables and depicted the information graphically (i.e., in an EGM). The structure of the tables follows the BANANA approach and provides a comprehensive overview of all relevant information. More detailed information on the assessed approaches can be found in the Additional files 4 and 5.

To provide an overview of contextual factors assessed, an EGM was developed using two software tools: EPPI-Reviewer Version 4.12.3.0 [39] and EPPI-Mapper Version 1.2.5 [40]. As terminology and conceptualization of contextual factors varied widely across the identified studies, with none differentiating between context and setting, we used the CICI framework to categorize identified micro-, meso- und macro-level aspects [2]. Contextual factors were grouped to the seven CICI context domains (i.e., geographical, epidemiological, socio-cultural, socio-economic, political, legal and ethical) and subcategories further specifying contextual domains (e.g., infrastructure, organization structure, leadership). Setting factors as part of the context (i.e., those referring to the physical location in which an intervention is implemented) were categorized into three domains: work environment, physical characteristics and practice patterns. Since included studies did not differentiated setting as a part of context, JM inductively categorized all identified setting factors for each domain (e.g., pertaining to work flow, capacity, availability of resources) to clearly structure and summarize them. These choices were then reviewed by TB. Inconsistencies were discussed with SDG and FZ. Using dots, the evidence map concisely depicts which aspects of context and setting were assessed in each implementation and at which level. Each dot's color indicates whether the method used was quantitative or qualitative; its size indicates how many studies investigated this aspect. I.e., the larger the dot, the more studies have considered this specific aspect. As the evidence map is interactive, categories can be shown or hidden to provide simpler or more complex views. The respective studies' references (including abstracts) can also be displayed.

### Evaluation of identified methodological approaches

To critically evaluate the methodological approaches to contextual analysis reported in the included studies, we grouped the extracted data via five of the six components described in the BANANA approach [27]. The sixth step of BANANA was not evaluated as it refers to the reporting of the contextual analysis, which was an inclusion criterion for the assessed studies. We applied color-coding to indicate whether each study clearly addressed a component (green), only mentioned it partly (yellow), or failed to address it (red). The color coding was done independently by two researchers (JM, TB). In cases of disagreement, a third researcher (SDG) was consulted to decide on the rating.

## Results

**Fig. 1:**
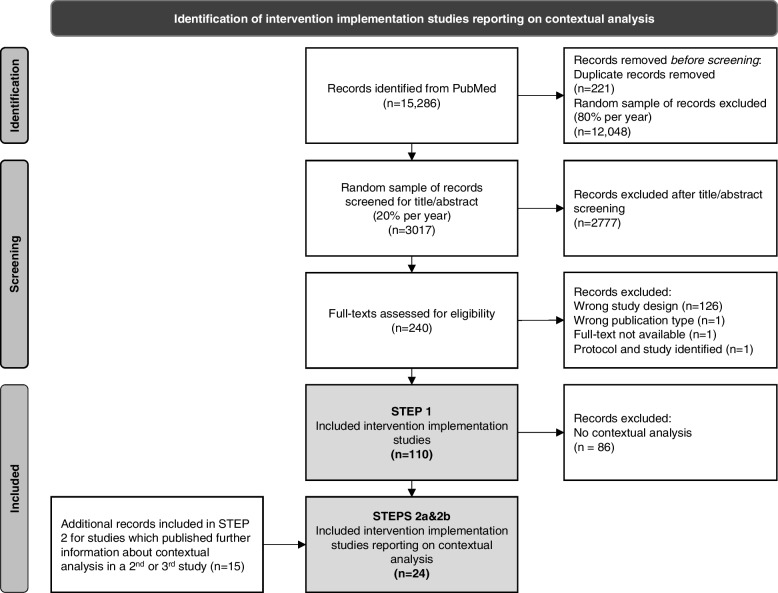
PRISMA flow chart presenting a stepwise approach to identify relevant studies

We used a two-phase sampling process. In Phase 1, our PubMed search returned 15,286 records. After removing duplicates, we randomly sampled 20% of the remaining studies from each of the six selected publication years (2015–2020) (n = 3017). In Phase 2, we screened this sample via the inclusion criteria noted above. Figure 1 presents a flow chart of the screening process. This left 110 intervention implementation studies for data extraction. For Phase 1, our inter-rater reliability was 76.7%; for Phase 2 it was 91.1%. As the included articles were both, original studies and study protocols, in the interests of readability, we will describe all results in the past tense.

### General characteristics of included studies (Step 1)

Of the 110 extracted articles the majority were study protocols (*n* = 90); most (*n* = 82) were either from North America (*n* = 45) or Europe (*n* = 37) (Table 1). The studies were conducted in a wide range of settings, the most common being primary care (*n* = 20), community care (*n* = 15), the health care system (*n* = 13) and schools (*n* = 12). Eighty-four of their designs were experimental; twenty-six were quasi-experimental. Further details of the studies are described in the Additional file 4.

**Table 1:** Characteristics of intervention implementation studies included in step 1 and step 2 in n (%)^1^

		Studies step 1 (*n* = 110)	Studies step 2 (*n* = 24)
Article type	Study protocol	90 (81.8)	22 (91.7)
	Original article	20 (18.2)	2 (8.3)
Continent	North America	45 (40.9)	11 (45.8)
	Europe	37 (33.6)	10 (41.7)
	Australia	14 (12.7)	2 (8.3)
	Africa	7 (6.4)	1 (4.2)
	Asia	5 (4.6)	-
	South America	2 (1.8)	-
Setting	Health care	72 (65.5)	16 (66.7)
	Primary care	20 (31.8)	5 (20.8)
	Health care system	13 (11.8)	4 (16.7)
	Hospitals	9 (8.2)	2 (8.3)
	Nursing homes	9 (8.2)	1 (4.2)
	Mental health care	7 (6.4)	-
	Outpatient care	5 (4.6)	1 (4.2)
	Emergency departments	4 (3.6)	2 (8.3)
	Rehabilitation services	3 (2.7)	1 (4.2)
	Veterans Health Administration	2 (1.8)	-
	Community settings	35 (31.8)	7 (29.2)
	Community care	15 (13.6)	1 (4.2)
	Schools	12 (10.9)	4 (16.7)
	Workplace	2 (1.8)	
	Churches	2 (1.8)	-
	Justice	2 (1.8)	2 (8.3)
	Kindergarten	2 (1.8)	-
	Other	3 (2.7)	1 (4.2)
	Family planning services	1 (0.9)	-
	Pharmacies	1 (0.9)	-
	Supermarkets	1 (0.9)	1 (4.2)
Study design testing clinical effectiveness	Experimental	84 (76.4)	17 (70.8)
	Quasi-experimental	26 (23.6)	7 (29.2)

Note. ^1^ Step 1 focusses on all identified intervention implementation studies, step 2 focusses only on studies that conducted a contextual analysis

### Characteristics of studies reporting on contextual analysis and methodological approaches applied (Step 2)

Of the sample's 110 studies, 24 (21.8%) reported conducting contextual analyses (Table 2). As authors of seven studies had released further information or results elsewhere, we located and extracted those records (*n* = 15) as well. Based on Curran et al.'s definitions [38], we identified (or categorized if not described) 17 hybrid type 1, five hybrid type 2, and two hybrid type 3 designs. Seven of the 24 assessed context at one time point; 12 assessed it at two, and five at three timepoints during their projects (Additional file 5).

**Table 2:** Overview of included studies conducting a contextual analysis

**Study**	**First author, (**^**1**^**)**	**Year**	**Article type**	**Country**	**Design**^2^(Outcomes see Additional file 5)	**Setting**	**Intervention**	**Intervention level**			**Process evaluation**^**3**^
								**Micro**	**Meso**	**Macro**	
1	Apers et al. [41]	2020	Study protocol	Belgium	Hybrid Type 1 CE: Modified stepped wedge cluster design I: Mixed-methods	Primary care	HIV-testing intervention in primary care (general practitioners)		X		Y
2	Berhanu et al. [42]	2020	Study protocol	Ethiopia	Hybrid Type 1 CE: Pre-post randomized control group design I: Mixed-methods	Primary care	Optimizing Health Extension Program to increase the utilization of primary child health services		X		Y
3	Bidwell et al. [43]	2018	Study protocol	UK	Hybrid Type 2 CE: Randomized stepped-wedge design I: Mixed methods	Hospitals	Care bundle to reduce incidence of obstetric anal sphincter injuries	X	X		N
4	D'Onofrio et al. [44, 45]	2019	Study protocol	USA	Hybrid Type 3 CE: Modified stepped wedge design I: Mixed methods	Emergency departments	Emergency department-initiated buprenorphine for patients with opioid use disorder	X	X		N
5	Grazioli et al. [46–49]	2019	Study protocol	Switzerland	Hybrid Type 2 CE: Randomized pre-post design I: Mixed methods	Emergency departments	Case management intervention for frequent users of the emergency department	X	X		Y
6	Hartzler et al. [50]	2017	Study protocol	USA	Hybrid Type 2 CE: Randomized controlled trial I: Mixed methods	Schools	Teen marijuana check-up in schools	X	X		N
7	Johnson et al. [51]	2018	Study protocol	USA	Hybrid Type 1 CE: Randomized controlled trial I: Mixed methods	Justice	Afrocentric, group-based, computerized HIV / sexually transmitted infection (STI) prevention intervention for controlled substance-using black women in community corrections settings	X			N
8	Knight et al. [52, 53]	2016	Study protocol	USA	Hybrid Type 3 CE: Cluster randomized interrupted time series I: Multi methods	Justice	Interventions for Adolescents in the Legal System for substance abuse		X	X	N
9	Kwan et al. [54]	2020	Study protocol	USA	Hybrid Type 1 CE: Cluster randomized controlled trial I: Mixed methods	Primary care	Patient-driven, shared medical appointments for providing diabetes self-management education and self-management support	X	X		N
10	Lakerveld et al. [55]	2018	Study protocol	Netherlands	Hybrid Type 1 CE: Randomized controlled trial I: Mixed methods	Supermarkets	Multi-level intervention using pricing and nudging strategies in the supermarket and context-specific mobile physical activity promotion app to impact on lifestyle behaviors and cardiometabolic health in adults with lower socio-economic status	X	X		N
11	Nahar et al [56]	2020	Study protocol	UK	Hybrid Type 1 CE: Stepped wedge cluster randomized controlled trial I: Mixed methods	Health care system	Multi-component community engagement intervention for cardiovascular disease prevention in socially disadvantaged populations	X	X		Y
12	Osilla et al. [57]	2020	Study protocol	USA	Hybrid Type 1 CE: Randomized controlled trial I: Survey	Outpatient care	Group-based therapy for support persons of adults on buprenorphine/naloxone to engage treatment resistant persons into treatment through positive communication and other behavioral strategies	X	X		U
13	Quintiliani et al. [58]	2015	Study protocol	USA	Hybrid Type 1 CE: Randomized controlled trial I: Multi methods	Primary care	Smoking-cessation intervention that combines patient navigation and financial incentives	X	X		Y
14	Rahm et al. [59]	2018	Study protocol	USA	Hybrid Type 1 CE: Unclear I: Qualitative and configurational comparative methodology	Health care system	Organizational toolkit for Lynch syndrome screening			X	Y
15	Rotter et al. [60]	2017	Study protocol	Canada	Hybrid Type 2 CE: Interrupted time series design with control groups I: unclear	Health care system	Clinical pathways for treatment of COPD		X		N
16	Saevareid et al. [61, 62–64]	2018	Study protocol	Norway	Hybrid Type 1 CE: Cluster randomized controlled trial I: Mixed methods	Nursing homes	Advanced care planning intervention in nursing homes		X		Y
17	Shanley et al. [65]	2019	Study protocol	Australia	Hybrid Type 2 CE: Pre-post design I: Mixed methods	Health care system	Innovative, tiered, culturally sensitive, neurodevelopmental assessment process within remote geographic locations with limited professional expertise, that considers fetal alcohol spectrum disorders as a potential outcome		X	X	Y
18	Smeltzer et al. [66, 67]	2018	Original article	USA	Hybrid Type 1 CE: Pre-post control group design I: unclear	Community care	Multidisciplinary lung cancer care model	X	X		Y
19	Steele Gray et al. [68–70]	2016	Study protocol	Canada	Hybrid Type 1 CE: Cluster randomized controlled trial I: Mixed methods	Primary care	Electronic patient reported (ePRO) mobile app and portal to creating and monitoring goal-oriented patient-care plans to improve patient self-management and shared decision making between patients and health care providers as well as proactive patient monitoring by the patient, caregiver(s), and health care provider	X	X		Y
20	Sutherland et al. ([71–73]; Janssen L, Sutherland R, Nathan N: Parent acceptability of using a mobile phone application, unpublished)	2019	Study protocol	Australia	Hybrid Type 1 CE: Cluster randomized controlled trial I: Mixed methods	Schools	Multi-component intervention that uses an existing school-based communication application to reduce kilojoule content from discretionary foods and drinks consumed by children from school lunch boxes whilst at school	X	X		N
21	Taylor et al. [74, 75]	2015	Original article	UK	Hybrid Type 1 CE: randomized controlled trial I: Multi methods	Rehabilitation services	Home-based self-care rehabilitation intervention in heart failure patients and caregivers	X	X		Y
22	van Delft et al. [76]	2019	Study protocol	Netherlands	Hybrid Type 1 CE: Pre-post design I: Mixed methods	Hospitals	Complex, multidimensional intervention to improve physical behavior during hospitalization, i.e., decrease patients` sedentary behavior and increase physical activity	X	X		Y
23	van Dongen et al. [77, 78]	2019	Study protocol	Netherlands	Hybrid Type 1 CE: Controlled time series design I: Mixed methods	Schools	Community based school intervention including four strategies for building the community capacity of students, school personnel, and parents	X	X		Y
24	Verjans-Janssen et al. [79]	2018	Study protocol	Netherlands	Hybrid Type 1 CE: Pre-post control group design I: Mixed mehods	Schools	School-based physical activity and nutrition intervention including family-based lifestyle parenting program	X	X		N

**Note:** ^1^reference of further paper(s) in which results of the contextual analysis were published in italics; ^2^CE = study design clinical effectiveness, I = study design implementation, RCT = randomized controlled trial; Hybrid Type 1 = prime focus on testing intervention effectiveness, and second, studying implementation. Hybrid Type 2 = equal focus on testing intervention effectiveness and implementation strategies; Hybrid Type 3 = prime focus on testing effectiveness of implementation strategies, and second, assessing the intervention; ^3^Process evaluation planned or results described as part of the intervention implementation study: Y = yes, N = not reported, U = unclear

### TMFs used and empirical evidence considered for contextual analysis

**Fig. 2:**
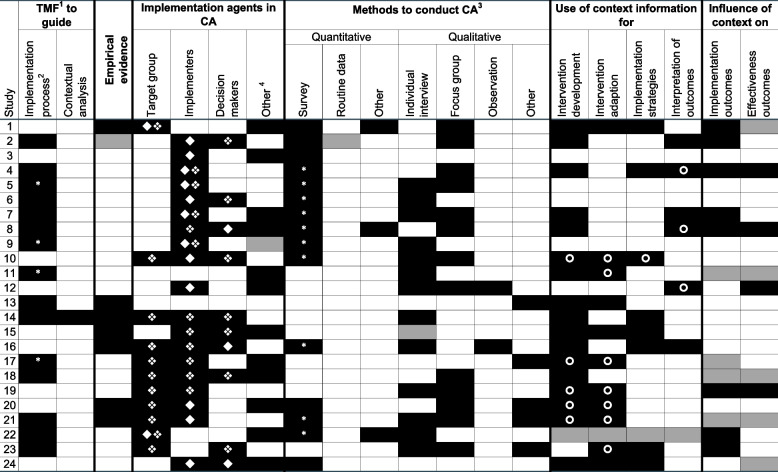
Characteristics of studies that performed contextual analyses (CAs) Note. Color coding: black = reported, white = not reported, grey = unclear; 1 TMF = theory, model, frameworks; 2 IP = overall implementation process in the assessed study, asterisk indicates combination of two TMFs; 3 asterisk indicates mixed methods analysis; 4 expert group / advisory panel; quantitative, qualitative; authors disescribed the process how contextual information were used

The included studies used eleven distinct TMFs. Those used can be broadly categorized into process models (e.g., Knowledge-to-Action Models), determinant frameworks (e.g., CFIR), or classic theories (e.g., social cognitive theory) [80]. One, the RE-AIM (reach, effectiveness, adoption, implementation, maintenance) Planning and Evaluation Framework is a process and evaluation framework that includes a determinant component [81]. Only one study specifically described how it used a TMF (CFIR) for contextual analysis and how that TMF guided it [59]. The others (*n* = 15) referred more generally to their TMFs guiding their overall implementation process, with RE-AIM (*n* = 7) and the Consolidated Framework for Implementation Research (CFIR) (*n* = 3), cited most often. Four studies reported combining two TMFs, e.g., CFIR and RE-AIM. In addition, seven considered empirical evidence about relevant contextual factors (Fig. 2).

### Consideration of implementation agents

Only nine studies collected data of all three types of implementation agents, with implementers most often being involved in the assessment of context (*n* = 19) (Fig. 2). In some cases, stakeholder groups who functioned as expert panels or advisory boards throughout the project (*n* = 11) were established. These included, e.g., health care providers from various medical fields, people affected by specific illnesses or health conditions, leaders and administrators, and delegates for non-profit organizations or government departments (Additional file 5).

### Methods applied for data collection and analysis

Of the 24 studies that reported using contextual analyses, 23 clearly described their methods. Of these 23, while ten explicitly reported using mixed-methods analysis, we found that 13 applied combinations of quantitative and qualitative methods. The remaining ten applied either quantitative (*n* = 2) or qualitative (*n* = 8) methods alone (Fig. 2). Quantitative data collection methods included purpose-designed surveys (*n* = 15), behavior mapping (*n* = 1), and retrospective use of national survey (*n* = 1) and surveillance (*n* = 1) data. Seven qualitative data collection methods were used: individual interviews (*n* = 13), focus groups (*n* = 13), observations (*n* = 2), as well as photovoice methodology^3^ (*n* = 2), telephone interviews (*n* = 1), yarning^4^ (*n* = 1) and site visits (*n* = 1).

### Contextual and setting factors assessed

We identified 43 separate factors. Following the CICI framework, we first categorized these as either context (*n* = 30) or setting factors (*n* = 13), then mapped them on an evidence gap map (Additional file 6) [2]. In general, meso-level factors (*n* = 22) were most commonly assessed, accounting for almost half of all mentions. The remainder were roughly equally divided between macro- (*n* = 13) and micro-level factors (*n* = 12). Fifteen studies considered context on at least two levels. We report a detailed overview of all assessed factors in Additional file 7.

*Contextual factors.* Within context, socio-cultural factors were most commonly assessed (e.g., knowledge and perceptions, lifestyle, social structure) (*n* = 20); no studies reported on legal aspects. In descending order of frequency, other contextual domains included political (e.g., policies, leadership) (*n* = 12), geographic (e.g., larger infrastructure) (*n* = 5), epidemiological (e.g., incidence and prevalence of disease) (*n* = 5), socio-economic (occupational aspects, living conditions) and ethical (ethical principles (*n* = 2), conflicts of interest (*n* = 2)). Seven studies described their assessment of inner or outer context or of facilitators and barriers, but did not further specify contextual factors in detail.

*Setting factors.* In view of setting, most studies assessments focused on the work environment (e.g., availability and accessibility of resources) (*n* = 15). Other setting aspects assessed included practice patterns (e.g., service delivery, care planning) (*n* = 11) as well as the setting's physical characteristics (e.g., study site, physical environment) (*n* = 7).

### Use of contextual information for subsequent project phases

Eighteen study protocols described further uses of contextual information to develop (*n* = 17) and/or adapt interventions (*n* = 11), eight used contextual information to choose implementation strategies, and six used it to interpret study outcomes. Of these, ten described their processes for doing that. Both original study papers described the further use of contextual information; however, only one reported how it was used.

### Influences of contextual factors on outcomes

Twelve study protocols and both original studies reported process evaluations. We identified nine studies that explicitly reported contextual factors' influences on implementation outcomes and/or effectiveness outcomes (Fig. 2). Various terms were used to signify similar implementation outcomes; and even where studies labeled these outcomes similarly, their definitions varied. In five protocol papers, as well as in both original studies, it was unclear whether any association had been considered between contextual factors and either implementation outcomes or effectiveness outcomes.

### Evaluation of methodological approaches for contextual analysis

**Fig. 3:**
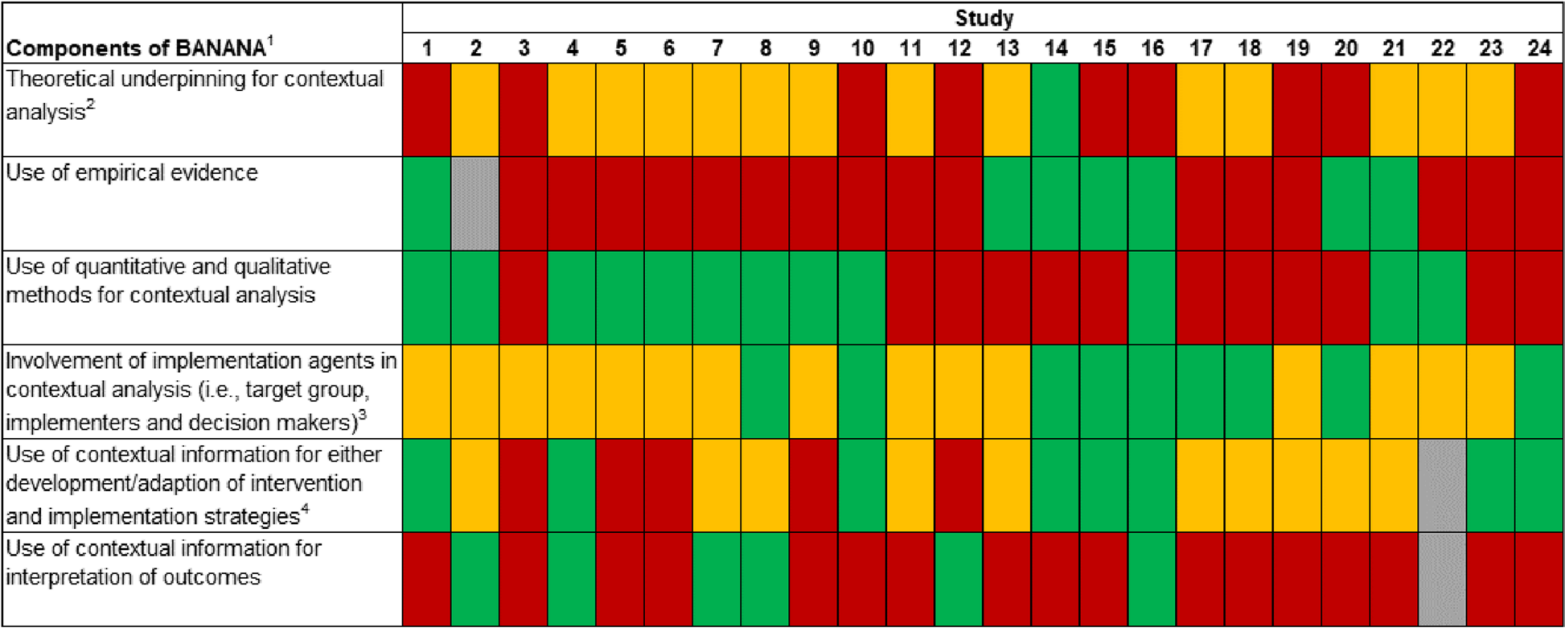
Evaluation of contextual analyses according to the Basel Approach for Contextual Analysis (BANANA) Note. Colors indicate, whether each study clearly addressed a component (green box), only mentioned it partly (yellow box), failed to address it (red box), or if it is unclear whether the component is addressed (grey box). 1 including components 1–5, whereas component five was divided into intervention/implementation strategies and implementation outcomes; as component six refers to the reporting of contextual analysis it is not included in this figure Further explanations on color codes of specific components: 2 green = TMF indicated to specifically guide contextual analysis, yellow = TMF indicated to guide overall implementation process, red = no TMF indicated; 3 green = all types of implementation agents were involved (i.e., target group, implementers, and decision makers), yellow = at least two types of implementation agents were involved, red = no involvement of implementation agents described; 4 green = use of contextual information for intervention and implementation strategy development/adaption, yellow = use for either intervention or implementation strategy development/adaption, red = use of contextual information not described; We have checked the colors used with the Chromatic Vision Simulator Web Edition 1.3 for their blind-friendliness

Our evaluation of the identified approaches to contextual analysis revealed that few studies addressed the key components of contextual analysis that are described in detail within BANANA (Fig. 3). The components that most studies clearly described were *the use of quantitative and qualitative methods* (*n* = 12) and *the involvement of implementation agents* (*n* = 9). The latter was also described *partly* within most of the remaining studies (*n* = 15). The two least addressed components were *the use of contextual information to interpret outcomes* (*n* = 7) and *the use of empirical evidence* (*n* = 7).

## Discussion

This study provides an overview of the current methodological approaches to contextual analysis in intervention implementation studies and indicates gaps. Using EGM methodology, we applied a novel approach for summarizing and evaluating available evidence on contextual analysis to develop an initial evidence gap map on contextual analysis methodology. Based on a random sample drawn from 110 intervention implementation studies, we found that fewer than one-quarter of those studies (21.8%) reported on analyses of their projects' contexts and settings. The studies that did report on contextual analyses showed high variability in the methodological approaches they used. This was true both of the analyses and of how they were reported.

Using the BANANA approach—one of the first frameworks for evaluating CAs—we found widespread significant methodological gaps. For example, few contextual analyses were theory based: only one study explicitly reported the use of a TMF for its contextual analysis; and fewer than half (8/22) provided information how they used findings from their CA to inform their project's subsequent steps.

### Lack of TMFs guiding contextual analysis

Building our understanding of context demands a stable theoretical basis. In addition to guiding our selection of multilevel contextual factors, this will enable operationalization both of context and of setting. Still, of the 24 studies we reviewed, only one provided both a specific description of its authors' use of a TMF to guide their contextual analysis and their rationale for using the one they did [59, 82]. Congruent with our findings, research shows that 22.5 – 48% of implementation science studies typically use TMFs; and of those that do, few explicitly explain their choices [82–85].

The phenomenon of “underuse, superficial use or misuse” [86] of TMFs has been described elsewhere in implementation science literature [85, 87–89]. All of the identified TMFs consider context, but differ widely regarding their focus and conceptualization of context [18, 20]. Lacking clear theoretical underpinnings, their assessments of contextual factors appear arbitrary. While limiting both the comparability and the generalizability of their results, this gives the impression of a lack of rigor concerning the contextual analysis. And as this analysis provides the data for further fine-tuning of the project, any such deficiencies will reduce the credibility of all subsequent study phases. This includes also the emerging focus of differentiating setting from context, which was not reflected in includes studies and complicated data analysis [2, 16].

### Variability in conceptualization and assessment of context

Consistent with other reviews' findings, the assessed studies' conceptualizations of context tended to be vague. For example, while a diverse range of factors were assessed at numerous levels, no definitions accompanied them. The resulting vagueness (e.g., documentation of inner and outer context, local contextual determinants, environmental-level characteristics, facilitators and barriers), hampered our efforts either to understand, to summarize and to compare those factors [13, 17, 18].

We noted considerable differences regarding which levels' and domains' contextual factors were appropriate targets for investigation. In contrast to Rogers et al.'s review [18] of studies from 2008–2018, which found that micro-level factors were most often assessed, our results regarding reports published over the last six years (2015–2020) showed a significant focus on the meso level, with socio-cultural contextual factors (e.g., social structure, community structure) most frequently captured. Macro-level factors (including political, legal and socio-economic aspects) were less commonly studied.

This scarcity might also reflect a shortage of tools and frameworks focusing on the macro level [20, 24, 90, 91]. However, evidence points to the importance of macro-level factors for adoption and successful implementation of interventions. For example, policy dynamics—or rather, competing policy agendas—can create major macro-level barriers to implementation [90, 92, 93]. Further, when reviewing research on projects that resulted in mis-implementation of interventions, it quickly becomes clear that the most common causes of premature termination of effective interventions or programs are all funding-related (86–87.6%) [94, 95]. This observation drives home the point that, to maximize the chances of a project's success (e.g., by recognizing changes in funding priorities at an early stage acquiring additional funding), its contextual analyses has to consider and closely monitor factors at every level.

However, the choice of which contextual factors to study and which stakeholders to involve at which phases depends largely on the type of intervention. This may also explain why the recorded contextual factors differed so widely between studies.

Furthermore, both the assessment of context and the reporting of contextual analysis might be biased by their analysts' level of pre-existing knowledge, i.e., researchers' inside knowledge may influence the quality or impartiality of their results. For example, researchers working in a specific setting may already be aware of certain contextual determinants (e.g., processes and practice patterns) or may gather important information informally (e.g., via chance meetings with implementation agents, observation of practice). While this information is not explicitly collected for the contextual analysis, it can lead to confirmation bias. I.e., it can leave "blind spots" in contextual analysis, exerting subtle pressure on analysis or interpretation to favor factors that support pre-existing hypotheses or beliefs [96].

### Limited involvement of various implementation agents

Both to enhance the quality of a project's research and to ensure appropriateness of intervention and implementation strategies through co-design, it is crucial to involve implementation agents in diverse positions [97, 98]. This is true throughout the implementation project but especially so in the contextual analysis. Also, in the reviewed studies, the most commonly considered implementation agents were implementers; however, persons affected by the intervention and decision makers often went unrepresented. Implementation science guidelines generally recommend the most representative possible range of implementation agents' and other stakeholders' voices—the clear assumption being that this improves the likelihood of a successful and sustainable implementation [99]. In order to benefit fully from implementation agents' views, a stakeholder involvement strategy should be developed, specifying both, the tasks performed by the involved implementation agents and the methods used to involve them [100].

### Variability in methods used for contextual analysis

For contextual analysis, either a combination of quantitative and qualitative methods, or, if possible, a mixed-methods approach is recommended. Merging, connecting or embedding data obtained via various means increases both the breadth and the depth of the analysis [101, 102]. It also improves our practical understanding of how interventions can work and of which implementation strategies are needed to successfully implement them [101, 103]. Congruent with Rogers et al.'s findings [18], we found that only 37.5% of the studies used mixed-methods approaches [104, 105]. Overall, while Rogers et al.'s sample included a smaller proportion of these approaches (19%), the tendency was the same. Like them, our sample also used more qualitative than quantitative methods (respectively 75% and 25% compared to Rogers et al.'s respective findings of 53% and 28%).

Likewise, surveys or interviews (with individuals or focus groups) were our sample's most common methods of capturing contextual details. However, recent studies increasingly emphasize the relevance of direct (e.g., ethnographic) observations in implementation research. These allow insider perspectives, including, for example, records of contextual aspects that implementation agents may take for granted and omit to mention, or tasks performed differently than generally reported [106–109].

Problematically, as contextual analysis in implementation science is primarily done within a postpositivist paradigm, researchers' understandings of context are often mechanistic and reductionistic. Therefore, we recommend that they also consider constructivist perspectives, particularly rapid ethnographic methods. In addition to probing more deeply into the context (e.g., to uncover hidden processes), these require fewer resources than traditional methods. This efficiency makes them particularly useful for contextual analyses, which are rarely well-resourced [108, 110, 111].

### Gaps in reporting and use of contextual information

As noted above, the reviewed studies showed significant gaps in their descriptions of how contextual information was later used. The results mainly informed intervention development. However, reporting gaps may have resulted from the fact that we assessed study protocols almost exclusively.

Another factor influencing the reporting of contextual analyses in study protocols or journal articles is lack of space: a 5000-word article can adequately develop and describe its central topic, but very little more. Therefore, implementation scientists should consider publishing their contextual analyses as separate papers. This would allow detailed descriptions of their methods and results, as well as of how they used those results for further study phases. Detailed reporting guidelines for contextual analysis could help researchers to structure their findings and avoid the types of “blind spots” noted above.

### Strengths and weaknesses

The current study's objective is to systematically review and map methodological approaches currently in use for contextual analyses, as well as to identify gaps in the identified approaches. In this regard, this paper's most notable strength is the empirical search string development. Given the reported challenges in finding implementation science literature, the string provides both high sensitivity and high specificity [112–114].

Furthermore, we provide a novel framework for evaluating existing CA-related evidence by applying the BANANA approach [27]. This framework can be used as a monitoring system for literature on contextual analysis, while providing quality criteria to evaluate contextual analysis. Moreover, the developed EGM offers a concise and informative overview of the reviewed studies' results, thereby facilitating comparison between them. The map is a “living document” designed to be updatable by future researchers.

However, as we included primarily study protocols, the descriptions given of contextual analysis lacked adequate detail in some cases. This affects our analysis of how contextual information informed the studied projects' later phases. Although we searched study papers related to the protocol, we were unable to verify in every case the extent to which the planned approaches to contextual analysis were carried out in the project, or whether adaptations were made. We suspect that one major reason for the high number of identified study protocols was publishing bias. Considering that we only included articles reporting contextual analyses as part of intervention implementation studies, it is possible that many contextual analyses were reported in study protocols, then conducted as part of implementation projects but not published.

The applied random sampling approach of study papers provided an opportunity to gain an initial overview of current evidence and its gaps. However, this approach may have excluded other relevant study papers that could have provided further insights into approaches to contextual analyses. Another possible weakness is that our strict inclusion criteria might have influenced our results. We focused on contextual analysis as a foundation for further study phases, i.e., prospective assessment of context and setting factors. As studies that conducted their contextual analyses retrospectively (e.g., as part of their process evaluation) would not enhance our understanding of contextual analysis in implementation science, we excluded them. For further research, it would be useful to adapt BANANA by planning a more comprehensive analysis—one that differentiates between the different implementation project phases (e.g., exploration, preparation, implementation and sustainment phase [115]). This would allow us to study differences in approaches applied to contextual analysis, that might be related to the different phases of an implementation project (e.g., contextual factors assessed might differ in the exploration and sustainment phase.

## Conclusions

To the best of our knowledge, this is the first study to provide a novel framework for evaluating and mapping methodological approaches to contextual analysis. Our evidence map provides a broad overview of methodologies applied in contextual analysis and shows which aspects of those studies can serve as models for other implementation science projects. The map is dynamic and can be updated as the literature on contextual analysis evolves.

We found wide variation regarding which methods were used for contextual analysis, which contextual factors were assessed, and how the results were applied in later study phases. Such a high level of heterogeneity is a major barrier to inter-study comparison or to later scale-up efforts. To reduce it, we recommend conducting contextual analyses according to TMFs. In addition to providing clear, proven and repeatable methodologies, these both support stronger conceptualization of the assessed context and enhance the rigor of the entire analytical process. If the described gaps are left open, contextual analysis will become a "black box" in many cases, greatly reducing its contribution over the course of implementation projects. Therefore, the implementation science community needs to take concerted action to develop, test and improve straightforward, robust methodologies for contextual analysis and reporting.

Across health care, researchers need to embrace contextual analysis as an essential element of every implementation science project; funding agencies need to develop specific opportunities to improve it; and journals need to demand full reporting on it. And every implementation science research team needs not only practical guidance on how to carry out contextual analysis, but also special guidelines on how to report their findings. Above all, we need to understand that, to achieve the quality and success that implementation science research promises, we will first need to break open the “black box” of contextual analysis.

## Supplementary Information

**Additional file 1. **Preferred Reporting Items for Systematic reviews and Meta-Analyses extension for Scoping Reviews (PRISMA-ScR) Checklist.

**Additional file 2. **Research questions and screening tool inclusion-/exclusion criteria.

**Additional file 3.** Empirical search string development.

**Additional file 4.** **STEP 1** - General characteristics of identified implementation intervention studies (*n* = 110).

**Additional file 5: STEP 2** - Study characteristics of implementation intervention studies that performed contextual analyses.

**Additional file 6.** Evidence gap map.

**Additional file 7: **Overview of contextual factors identified in implementation intervention studies (mapped according to the Context and Implementation of Complex Interventions (CICI) framework (1)).

## Data Availability

All data generated or analyzed during this study are included in this published article as supplementary information files.

## Abbreviations

BANANA: Basel Approach for coNtextual ANAlysis
CICI: Context an Implementation of Complex Interventions (CICI) framework
EGM: Evidence gap map
TMF: Theory, model or framework

## Footnotes

1 Implementation science is a scientific study, promoting “the systematic uptake of research findings and other evidence-based practices into routine practice, and, hence, to improve the quality and effectiveness of health services and care” [116].

2 Hybrid Type 1: Prime focus on testing intervention effectiveness, and second, studying implementation. Hybrid Type 2: equal focus on testing intervention effectiveness and implementation strategies. Hybrid Type 3: Prime focus on testing effectiveness of implementation strategies, and second, assessing the intervention.

3 Photovoice is a participatory research methodology, that allows study participants to record and reflect on their experiences (e.g., quit smoking) by taking photos and discussing those in guided discussion sessions [117, 118].

4 Yarning is a highly structured qualitative research methodology, to gain knowledge from indigenous people by storytelling [119].
