# Supplementary material for: Methodological approaches to study context in intervention implementation studies: an evidence gap map
Source: BMC Med Res Methodol. 2022 Dec 14;22:320. doi: 10.1186/s12874-022-01772-w (PMC9749183; doi:10.1186/s12874-022-01772-w)
Supplement: Supplementary file 2 — Additional file 2. Research questions and screening tool inclusion-/exclusion criteria. [file 12874_2022_1772_MOESM2_ESM.docx]

**Additional file 2: Research questions and screening tool inclusion-/exclusion criteria**

**Research questions**

| **Step 1 – Research questions with focus on all identified implementation intervention studies (limited to general data on the manuscript)** |
| --- |
| - What are the general characteristics of the studies regarding publication year, publishing journal, first author, research sector, geographical locations (country) of the research sites and design published journal? |
| - What are the geographical locations (country) of the research sites where the implementation intervention studies were conducted? |
| - Which hybrid designs were applied in the implementation intervention studies? |
| - What is the total number of implementation intervention studies conducting a contextual analysis? |
| **Step 2a - Research questions with focus on implementation intervention studies that performed a contextual analysis** |
| - In which phase of the implementation project were contextual factors assessed (preparatory phase, baseline, midpoint, post) and how was information gathered through contextual analysis used? |
| - Which theory, model or theoretical framework(s) according to Nilsen [26] guided the contextual analysis? |
| - Which contextual factors were assessed at which level(s) (micro, meso, macro) in the contextual analysis and does the level correspond to the level the intervention targets? |
| - Which domains of context were assessed in the contextual analysis according to the Context and Implementation of Complex interventions (CICI) framework [1]? |
| - Which methods were applied to conduct the contextual analysis? |
| - Was existing evidence from other empirical studies about relevant contextual factors considered in the contextual analysis? |
| - Were implementation agents or other stakeholders involved during the contextual analysis? |
| **Step 2b - Research questions that go beyond methods for conducting context analysis, but focus on the results of context analysis** |
| - For which further study phases (e.g. intervention development, choice of implementation strategies) were contextual information used and how? |
| - Which influences of contextual factors on implementation and effectiveness outcomes were reported? |

**Screening tool inclusion-/exclusion criteria**

*(All criteria needed to apply!)*

| **Step 1** | | |
| --- | --- | --- |
| ***Inclusion criteria*** | | |
|  | **Criterion** | **Explanation** |
| **1** | **Article type**  Peer-reviewed article or study protocol | Editorials, letters to the editor, commentaries, guidelines, conference abstracts, case reports and dissertation will be excluded. |
| **2** | **Study design**  Implementation intervention study | - Study tests effectiveness of an intervention (experimental or quasi-experimental design). |
|  |  | - Intervention tested entails one of the 7 Ps (programs, practices, principles, procedures, products, pills, and policies) [2]. |
|  |  | - Intervention tested will be implemented in daily (clinical) practice. |
|  |  | - Study reports on evaluation of implementation pathway (gathering information on implementation process qualitatively or quantitatively) and/or assessing implementation outcomes as defined by outcomes of Proctor et al. [3]). If feasibility is reported as implementation outcome, at least one further implementation outcome (e.g., acceptability) needs to be reported. |
| **3** | **Language** | Paper is written in English or German. |
| **4** | **Full text** | Full text is available. |
| ***Exclusion criteria*** | | |
| **5** | **Process evaluation** | Article is clearly labeled as process evaluation (title/abstract). |
| **6** | **Study protocol of an included study** | If study protocol: The study belonging to the protocol has already been included. |
| **Step 2** | | |
| ***Inclusion criteria*** | | |
| **7** | **Reports on contextual analysis** | - CA entails **quantitative, qualitative, or mixed-methods** information about the context and the setting, in which the intervention will be implemented. |
|  |  | - **Context** is defined as “a set of characteristics and circumstances that consist of active and unique factors, within which the implementation is embedded. As such, context is not a backdrop for implementation, but interacts, influences, modifies and facilitates or constrains the intervention and its implementation. Context is usually considered in relation to an intervention, with which it actively interacts. It is an overarching concept, comprising not only a physical location but also roles, interactions and relationships at multiple levels” [1]. |
|  |  | - CA is a separate step in an implementation intervention study (active data collection), which entails assessment of contextual information, e.g., **practice patterns, facilitators and barriers.** |
|  |  | - CA was done **prior to the start or throughout of the implementation intervention study** to inform, for example, intervention development or choice of implementation strategies. |
| ***Exclusion criteria*** | | |
|  | **Process evaluation** | - CA conducted only part of a **process evaluation** will be excluded |

**Definitions of implementation outcomes**

| **Table 1.** Implementation outcomes defined by Proctor et al. 2011 [3] | | |
| --- | --- | --- |
| **Implementation outcome** | **Definition [4]** | **Related terms** |
| Acceptability | Extent to which implementation stakeholders perceive a treatment, service, practice, or innovation to be agreeable, palatable, or satisfactory. | Satisfaction with various aspects of the innovation (e.g., content, complexity, comfort, delivery, and credibility) |
| Adoption | Intention, initial decision, or action to try or employ an innovation or evidence-based practice. | Uptake, utilization, initial implementation, intention to try |
| Appropriateness | Perceived fit, relevance, or compatibility of the innovation or evidence-based practice for a given practice setting, provider, or consumer; and/or perceived fit of the innovation or evidence-based practice to address a particular issue or problem. | Perceived fit, relevance. Compatibility, suitability, usefulness, practicability |
| Feasibility | Extent to which a new innovation or practice can be successfully used or carried out within a given agency or setting.  Reach + fidelity + dose = feasibility | Actual fit or utility, suitability for everyday use, practicability |
| Fidelity | Degree to which an intervention or implementation strategy was delivered as prescribed in the original protocol or as intended by program developers. May include multiple dimensions such as content, process, exposure, and dosage. | Delivered as intended, adherence, integrity, quality of program delivery |
| Implementation cost | Financial impact of an implementation effort. May include costs of treatment delivery, costs of the implementation strategy, and cost of using the service setting. | Marginal cost, cost-effectiveness,  cost-benefit |
| Penetration | Extent to which an innovation or practice is integrated | Level of institutionalization, spread, service access |
| Sustainability | Extent to which a recently implemented practice is maintained and / or institutionalized within a service setting`s ongoing, stable operations. | Maintenance, continuation, durability; incorporation, integration, institutionalization, sustained use, routinization |

**Data extraction tool**

| **Step 1** | | | |
| --- | --- | --- | --- |
|  | **Variable** | **Explanation** | **Extracted data** |
| **General information article** | **First author** | Record name of 1^st^ author |  |
|  | **Year** | Record year of publication |  |
|  | **Study title** | Record article title |  |
|  | **Journal** | Record name of the journal in which the article was published |  |
|  | **Article type** | Record type of included article | □ Study protocol  □ Original article |
|  | **Country** | Geographical location of study setting |  |
|  | **Setting** | Setting in which research was conducted |  |
| **Design** | **Hybrid Design** | Which hybrid design was applied for the overall intervention study? | □ Hybrid Type 1  □ Hybrid Type 2  □ Hybrid Type 3  □ Unclear |
|  | **Design clinical effectiveness** | Which design was applied to evaluate effectiveness? | □ Experimental  □ Quasi-experimental  □ Unclear |
|  | **Design implementation** | Which design was applied to evaluate implementation? |  |
| **Outcomes** | **Primary effectiveness outcome** | Record primary effectiveness outcome assessed |  |
|  | **Implementation outcome** | Record implementation outcome assessed |  |
| **Contextual analysis** | **Contextual analysis** | Does the study report on a contextual analysis? | □ Yes  □ No |
|  | **Information about contextual analysis published in a further article** | Is further information about the CA provided in a 2nd article? If yes record author, year and title | □ Yes  □ No  □ Unclear  Further information: |

| **Step 2** | | | |
| --- | --- | --- | --- |
|  | **Variable** | **Explanation** | **Extracted data** |
| **Intervention** | **Intervention** | Provide a brief description of the intervention |  |
|  | **Intervention level** | Indicate the level(s) the intervention targets | □ Micro  □ Meso  □ Macro |
| **Contextual analysis** | **Timepoint contextual analysis** | Record timepoint(s) at which contextual analysis was conducted | □ Preparatory phase  Implementation phase  □ Baseline  □ Midpoint  □ Post  □ Process evaluation |
|  | **Theory, model or framework** | Indicate theory, model or framework (TMF) applied to guide implementation process. |  |
|  |  | Indicate which theory, model or framework (TMF) was applied for contextual analysis. |  |
|  | **Levels of context assessed** | Indicate the level(s) at which context was studied | □ Micro  □ Meso  □ Macro |
|  | **Empirical evidence** | Was existing evidence from other empirical studies about relevant contextual factors considered in the contextual analysis? | □ Yes  □ No  □ Unclear |
|  | **Design** | Which study design was applied for contextual analysis? | □ Quantitative  □ Qualitative  □ Quantitative & qualitative  □ Unclear |

|  | **Variable** | **Explanation** | **Extracted data** |
| --- | --- | --- | --- |
|  | **Mixed methods** | Were data triangulated? | □ Yes  □ No  □ Unclear |
|  | **Methods (quantitative)** | Report which contextual factors were assessed quantitatively and indicate at which level factors were assessed (micro, meso, macro) | □ Unclear  □ Not reported |
|  |  | Specify quantitative methods applied to assess contextual factors | □ Survey  □ Routine data  □ Other:  □ Unclear  □ Not reported |
|  |  | Please indicate the measurement tool(s) used to assess contextual factors. | □ Tool validated  □ Tool self-developed  □ Both  □ Unclear  □ Not reported |
|  | **Methods (qualitative)** | Report which aspects of context were explored qualitatively and indicate level at which aspect were assessed (micro, meso, macro) | □ Unclear  □ Not reported |
|  |  | Specify qualitative data collection methods used for exploring context. | □ Individual interviews  □ Focus group interviews  □ Observation  □ Other: |

|  | **Variable** | **Explanation** | **Extracted data** |
| --- | --- | --- | --- |
|  | **Implementation agents and other stakeholders** | Indicate which implementation agents were included to assess contextual factors quantitatively? | Target group:  Implementers:  Decision makers:  Other: |
|  |  | Indicate which implementation agents were included to assess contextual factors qualitatively? | Target group:  Implementers:  Decision makers:  Other: |
|  |  | Was there a group of experts or an advisory board involved during the implementation study?  If yes, report participants. | □ Yes  □ No  □ Unclear  Participants: |
|  | **Funding** | Indicate whether funding was received for the study. | □ Yes, for overall project  □ Yes, specifically for CA  □ No  □ Unclear |

|  | **Variable** | **Explanation** | **Extracted data** |
| --- | --- | --- | --- |
| **Use of context information** | **Use of context information** | For which further study phase did authors used results from CA? | Intervention development  □ Yes  □ No  □ Unclear  Intervention adaption  □ Yes  □ No  □ Unclear  Implementation strategies  □ Yes  □ No  □ Unclear  Interpretation of outcomes  □ Yes  □ No  □ Unclear |
|  | **How context information was used** | Is there any description of the process how results were used to inform next study phase? | Intervention development  □ Yes  □ No  □ Unclear  Intervention adaption  □ Yes  □ No  □ Unclear  Implementation strategies  □ Yes  □ No  □ Unclear  Interpretation of outcomes  □ Yes  □ No  □ Unclear |

|  | **Variable** | **Explanation** | **Extracted data** |
| --- | --- | --- | --- |
| **Evaluation implementation pathway** | **Process evaluation** | Was a process evaluation planned or results of a process evaluation described as part of the implementation intervention study? | □ Described  □ Planned  □ Unclear  □ Not reported |
|  | **Influence on outcomes assessed** | Was an association between contextual factors and implementation outcomes assessed? | □ Yes  □ Planned  □ Unclear  □ Not reported |
|  |  | Was an association between contextual factors and summative outcomes assessed? | □ Yes  □ Planned  □ Unclear  □ Not reported |

**References**

1. Pfadenhauer LM, Gerhardus A, Mozygemba K, Lysdahl KB, Booth A, Hofmann B, Wahlster P, Polus S, Burns J, Brereton L *et al*: **Making sense of complexity in context and implementation: the Context and Implementation of Complex Interventions (CICI) framework**. *Implementation Science* 2017, **12**(1):21.

2. Brown CH, Curran G, Palinkas LA, Aarons GA, Wells KB, Jones L, Collins LM, Duan N, Mittman BS, Wallace A *et al*: **An Overview of Research and Evaluation Designs for Dissemination and Implementation**. *Annual Review of Public Health* 2017, **38**(1):1-22.

3. Proctor E, Silmere H, Raghavan R, Hovmand P, Aarons G, Bunger A, Griffey R, Hensley M: **Outcomes for implementation research: conceptual distinctions, measurement challenges, and research agenda**. *Administration and Policy in Mental Health and Mental Health Services Research* 2011, **38**(2):65-76.

4. Gerke D, Lewis E, Prusaczyk B, Hanley C, Baumann A, Proctor E: **Implementation Outcomes**. In: *Eight toolkits related to Dissemination and Implementation.* St. Louis: MO: Washington University; 2017.
