# Supplementary material for: Methodological approaches to study context in intervention implementation studies: an evidence gap map
Source: BMC Med Res Methodol. 2022 Dec 14;22:320. doi: 10.1186/s12874-022-01772-w (PMC9749183; doi:10.1186/s12874-022-01772-w)
Supplement: Supplementary file 3 — Additional file 3. Empirical search string development. [file 12874_2022_1772_MOESM3_ESM.docx]

**Additional file 3: Empirical search string development**

**Search string in Pubmed to identify relevant articles for development- and validation set (18.01.2021):**

implementation[Title/Abstract] OR adoption[Title/Abstract] OR dissemination[Title/Abstract] OR implementation research[Title/Abstract] OR complex intervention[Title/Abstract]

**AND**

acceptability[Title/Abstract] OR adoption[Title/Abstract] OR uptake[Title/Abstract] OR utilization[Title/Abstract] OR initial implementation[Title/Abstract] OR intention to try[Title/Abstract] OR appropriateness[Title/Abstract] OR perceived fit[Title/Abstract] OR relevance[Title/Abstract] OR compatibility[Title/Abstract] OR suitability[Title/Abstract] OR usefulness[Title/Abstract] OR practicability[Title/Abstract] OR feasibility OR utility[Title/Abstract] OR fidelity[Title/Abstract] OR adherence[Title/Abstract] OR integrity[Title/Abstract] OR implementation cost[Title/Abstract] OR penetration[Title/Abstract] OR sustainability[Title/Abstract] OR maintenance[Title/Abstract] OR continuation[Title/Abstract] OR durability[Title/Abstract] OR incorporation[Title/Abstract] OR integration[Title/Abstract] OR institutionalization[Title/Abstract] OR sustained use[Title/Abstract] OR routinization[Title/Abstract]

**AND**

study protocol[Title/Abstract] OR Clinical Trial Protocol[Publication Type] Filters: Clinical Trial, Clinical Trial Protocol, from 2006 - 2021 Sort by: Publication Date

**Screening of relevant articles to be included in the development and validation set:**


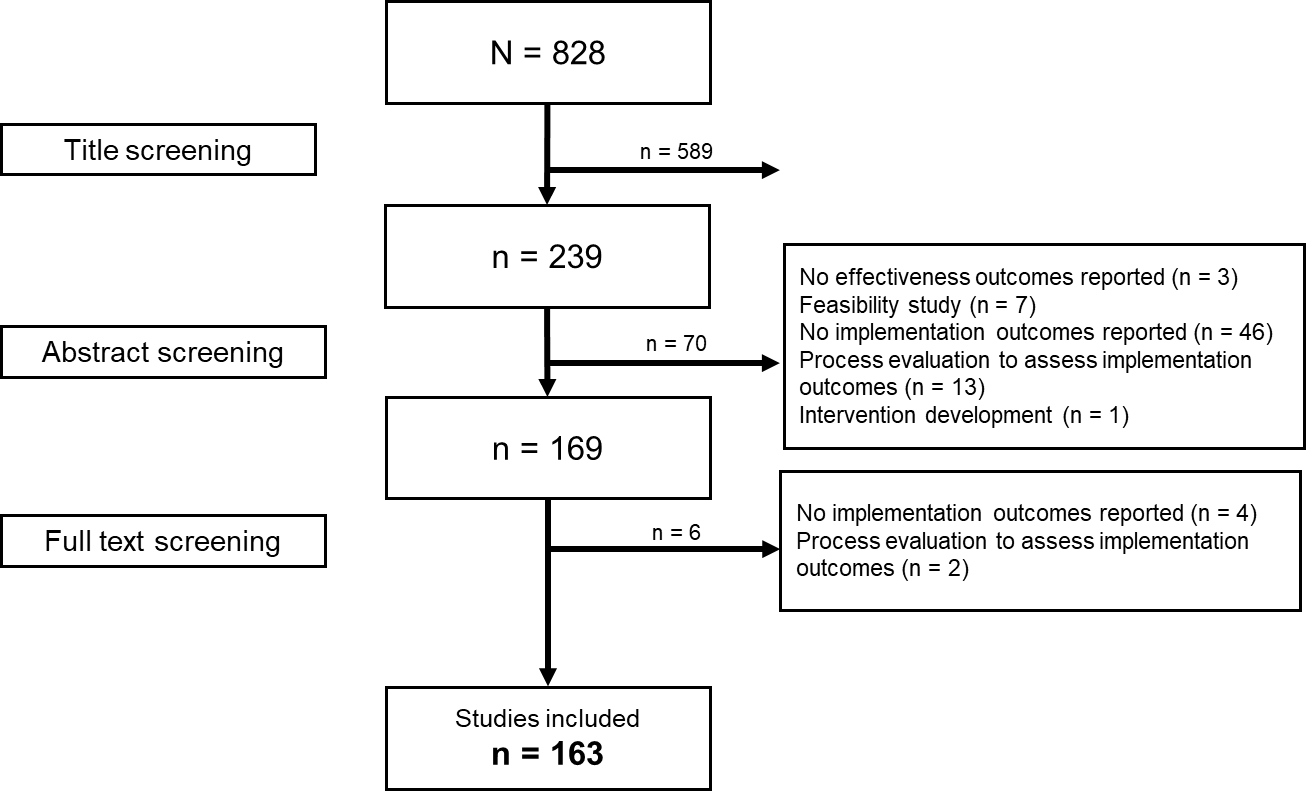


**Articles included in the development and validation set:**

Development Set (n = 81)

32552833 [PMID] OR 32160891 [PMID] OR 32075677 [PMID] OR 31996189 [PMID] OR 31907017 [PMID] OR 31882012 [PMID] OR 31842963 [PMID] OR 31791285 [PMID] OR 31753004 [PMID] OR 31712337 [PMID] OR 31678952 [PMID] OR 31594892 [PMID] OR 31500661 [PMID] OR 31477140 [PMID] OR 31310353 [PMID] OR 31409421 [PMID] OR 31326936 [PMID] OR 31307535 [PMID] OR 31289075 [PMID] OR 31248932 [PMID] OR 31233859 [PMID] OR 31196137 [PMID] OR 31092643 [PMID] OR 31046695 [PMID] OR 30951837 [PMID] OR 30904839 [PMID] OR 30872550 [PMID] OR 30798294 [PMID] OR 30772862 [PMID] OR 30683155 [PMID] OR 30634955 [PMID] OR 30782696 [PMID] OR 30604208 [PMID] OR 30594236 [PMID] OR 30576842 [PMID] OR 30552244 [PMID] OR 30514378 [PMID] OR 30290276 [PMID] OR 30103776 [PMID] OR 30075806 [PMID] OR 30055336 [PMID] OR 30021547 [PMID] OR 30005705 [PMID] OR 29895651 [PMID] OR 29880047 [PMID] OR 29866729 [PMID] OR 29788996 [PMID] OR 29764876 [PMID] OR 29739384 [PMID] OR 29661178 [PMID] OR 29606129 [PMID] OR 29522897 [PMID] OR 29374672 [PMID] OR 29370829 [PMID] OR 29288172 [PMID] OR 29157275 [PMID] OR 28821264 [PMID] OR 28630086 [PMID] OR 28166816 [PMID] OR 28109247 [PMID] OR 27884169 [PMID] OR 27798024 [PMID] OR 27756281 [PMID] OR 27592122 [PMID] OR 27553492 [PMID] OR 27473180 [PMID] OR 27400657 [PMID] OR 27130272 [PMID] OR 26936623 [PMID] OR 26831332 [PMID] OR 26353825 [PMID] OR 26268221 [PMID] OR 26297321 [PMID] OR 26100026 [PMID] OR 25873044 [PMID] OR 25527071 [PMID] OR 25273854 [PMID] OR 24950708 [PMID] OR 24559178 [PMID] OR 23758974 [PMID] OR 21851643 [PMID]

Validation Set (n = 82)

33419461 [PMID] OR 32375741 [PMID] OR 32084445 [PMID] OR 32005137 [PMID] OR 31907074 [PMID] OR 31888941 [PMID] OR 31874872 [PMID] OR 31829186 [PMID] OR 31784436 [PMID] OR 31727650 [PMID] OR 31699046 [PMID] OR 31619250 [PMID] OR 31525489 [PMID] OR 31481370 [PMID] OR 31462477 [PMID] OR 31416439 [PMID] OR 31345971 [PMID] OR 31324682 [PMID] OR 31300028 [PMID] OR 31289060 [PMID] OR 31248921 [PMID] OR 31215468 [PMID] OR 31138165 [PMID] OR 31063870 [PMID] OR 30992293 [PMID] OR 30928927 [PMID] OR 30898129 [PMID] OR 30808379 [PMID] OR 30777122 [PMID] OR 30696686 [PMID] OR 30782719 [PMID] OR 30782749 [PMID] OR 30630108 [PMID] OR 30598489 [PMID] OR 30587235 [PMID] OR 30576841 [PMID] OR 30547745 [PMID] OR 30383672 [PMID] OR 30208344 [PMID] OR 30081967 [PMID] OR 30071866 [PMID] OR 30041598 [PMID] OR 30007924 [PMID] OR 30146493 [PMID] OR 29884164 [PMID] OR 29866736 [PMID] OR 29858405 [PMID] OR 29769080 [PMID] OR 29739358 [PMID] OR 29716605 [PMID] OR 29625599 [PMID] OR 29602847 [PMID] OR 29506563 [PMID] OR 29373993 [PMID] OR 29334983 [PMID] OR 29202867 [PMID] OR 29078810 [PMID] OR 28720140 [PMID] OR 28532439 [PMID] OR 28115006 [PMID] OR 28003294 [PMID] OR 27842539 [PMID] OR 27770819 [PMID] OR 27707836 [PMID] OR 27557641 [PMID] OR 27473371 [PMID] OR 27417199 [PMID] OR 27354070 [PMID] OR 27084667 [PMID] OR 26845030 [PMID] OR 27015913 [PMID] OR 26345270 [PMID] OR 26223232 [PMID] OR 26112224 [PMID] OR 26018048 [PMID] OR 25887849 [PMID] OR 25443043 [PMID] OR 25224756 [PMID] OR 24719431 [PMID] OR 23924263 [PMID] OR 23731594 [PMID] OR 17274807 [PMID]

**Final search string:**

implementation science[MeSH Terms] OR implement[Title/Abstract] OR implementation[Title/Abstract] OR introduce[Title/Abstract] OR introduced[Title/Abstract] OR introducing[Title/Abstract] OR introduction[Title/Abstract]

**AND**

sustainable[Title/Abstract] OR sustainability[Title/Abstract] OR dissemine[Title/Abstract] OR dissemination[Title/Abstract] OR adherent[Title/Abstract] OR adherence[Title/Abstract] OR acceptable[Title/Abstract] OR acceptability[Title/Abstract] OR feasible[Title/Abstract] OR feasibility[Title/Abstract] OR feasibly[Title/Abstract] OR effectiveness[Title/Abstract]

**AND**

trial[Title/Abstract] OR trialing[Title/Abstract] OR trials[Title/Abstract]

Sensitivity development set: 95.1% (n=77)

Sensitivity validation set: 91.5% (n=75)
