# Supplementary material for: Methodological approaches to study context in intervention implementation studies: an evidence gap map
Source: BMC Med Res Methodol. 2022 Dec 14;22:320. doi: 10.1186/s12874-022-01772-w (PMC9749183; doi:10.1186/s12874-022-01772-w)
Supplement: Supplementary file 6 — Additional file 6. Evidence gap map. [file 12874_2022_1772_MOESM6_ESM.html]

EPPI-Mapper


X

- Filters
- Hide Headers
  Show Headers
- Fullscreen
  Exit Fullscreen
- About
- Submit a Study
- View Records

Contextual and setting factors assessed in implementation intervention studies (n=24)

Generated using v.2.1.0 of the EPPI-Mapper
powered by EPPI Reviewer
and created with


by the
Digital Solution Foundry team.
