# Supplementary material for: Methodological approaches to study context in intervention implementation studies: an evidence gap map
Source: BMC Med Res Methodol. 2022 Dec 14;22:320. doi: 10.1186/s12874-022-01772-w (PMC9749183; doi:10.1186/s12874-022-01772-w)
Supplement: Supplementary file 7 — Additional file 7: Overview of contextual factors identified in implementation intervention studies (mapped according to the Context and Implementation of Complex Interventions (CICI) framework (1)). [file 12874_2022_1772_MOESM7_ESM.docx]

**Additional file 7**

Overview of contextual factors identified in implementation intervention studies (mapped according to the Context and Implementation of Complex Interventions (CICI) framework (1)) *Qualitatively assessed; quantitatively assessed*

| **CONTEXT** | | | | | | | | | | | | | |
| --- | --- | --- | --- | --- | --- | --- | --- | --- | --- | --- | --- | --- | --- |
| **Domain** | **Level** | | | | | | | | | | | | |
|  | **Micro-level** | | | | **Meso-level** | | | | **Macro-level** | | | | |
|  | **Factor according CICI** | **Category defined** | **Factor identified** | **Ref** | **Factor according CICI** | **Category defined** | **Factor identified** | **Ref** | **Factor according CICI** | **Category defined** | **Factor identified** | **Ref** |  |
| **Geographical** | Infrastructure | Active transportation | | (2) | Infrastructure | *Relevant health services available* | | (3) | Infra-structure | District resources and infrastructure | | (4) |  |
|  |  |  |  |  |  |  |  |  |  | Range and number of treatment services in the emergency department catchment area | | (5) |  |
|  |  |  |  |  |  |  |  |  | General | *Questions that focus on the external environment of all recruitment and intervention locations* | | (6) |  |
| **Epidemiological** | Determinants of needs | *Patient needs and resources*  *Caregiver needs*  Support needs  *Current situation needs and desires on themes of community and family involvement*  *Support needs* | | (7)  (8)  (8)  (9)  (8) | - | | | | Demo-graphic | Demography | | (4) |  |
|  |  |  |  |  |  |  |  |  | Incidence/prevalence of disease and severity of disease | Number of undiagnosed HIV infections | | (10) |  |
|  |  |  |  |  |  |  |  |  |  | Prevalence of HIV | | (10) |  |
|  |  |  |  |  |  |  |  |  |  | Time distribution between HIV infection and diagnosis | | (10) |  |
|  |  |  |  |  |  |  |  |  |  | Changes over time | Recent epidemics and natural disasters | (4) |  |
| **Socio-cultural** | Knowledge and perceptions; beliefs | *Lynch syndrome knowledge and beliefs, perceptions of evidence*  *Opinion of exercise in general*  *Individual readiness to implement screening*  *Coming to terms with heart failure (incl. problems associated with the condition and how they were resolved)*  *GP`s perception of target group-based HIV testing*  Knowledge of disease and danger signs  Knowledge and beliefs of the intervention, that might influence adoption  Information needs  Sources of knowledge | | (7)  (11)  (7)  (8)  (10)  (4)  (6)  (8)  (4)  (4) | Social structure | Networks | *Organization structure*  Relational coordination and relationships in particular work processes in primary care teams  Networks | (7)  (12)  (6) | Know-ledge & perceptions | *Current community understanding of fetal alcohol spectrum disorder* | | (13) |  |
|  |  |  |  |  |  |  |  |  | Life-style | *The extent to which an unhealthy lifestyle as a risk factor for cardiometabolic health presents a societal problem*  *Lifestyle context of the community* | | (14)  (15) |  |
|  |  |  |  |  |  | Supervision and mentorship | Supportive supervision and mentorship from health centers to health posts | (4) | Social capital | Community engagement  *Community strengths and needs* | | (4)  (13) |  |
|  |  |  |  |  |  | Communication | Communication | (6, 12) |  |  |  |  |  |

| **CONTEXT** | | | | | | | | | | | | |
| --- | --- | --- | --- | --- | --- | --- | --- | --- | --- | --- | --- | --- |
| **Domain** | **Level** | | | | | | | | | | | |
|  | **Micro-level** | | | | **Meso-level** | | | | **Macro-level** | | | |
|  | **Factor according CICI** | **Category defined** | **Factor identified** | **Ref** | **Factor according CICI** | **Category defined** | **Factor identified** | **Ref** | **Factor according CICI** | **Category defined** | **Factor identified** | **Ref** |
| **Socio-cultural** | Family structures | Members of the household | | (4) | Social structure | Climate | Attitudes towards their workplace (organizational climate) | (16) | Social structure | Networks | *Systems map of case-processing in their jurisdiction depicting "linkages" with community behavioral health partners*  Relationships between JJ and BH agencies in providing services | (16)  (16) |
|  |  |  |  |  |  |  | Provider motivation | (17) |  |  |  |  |
|  |  |  |  |  |  |  | Commitment to work | (4) |  |  |  |  |
|  |  |  |  |  |  |  | Climate | (6) |  |  |  |  |
|  |  |  |  |  | Social structure | Culture | School culture | (9) |  |  |  |  |
|  |  |  |  |  |  |  | Practice culture | (12) |  |  |  |  |
|  |  |  |  |  |  |  | Work culture | (4) |  |  |  |  |
|  |  |  |  |  |  |  | Culture | (6) |  |  |  |  |
|  |  |  |  |  | Community characteristic and level of coordination/ involvement with community | Readiness for change | Organizational readiness for change | (5, 18, 19) |  |  |  |  |
|  |  |  |  |  |  |  | Whether they would consider changing app providers and what would encourage such a change | (20) |  |  |  |  |
|  |  |  |  |  |  | Implementation climate | Willingness to implement new practices | (17) |  |  |  |  |
|  |  |  |  |  |  |  | *Implementation climate in organization* | (7) |  |  |  |  |
|  |  |  |  |  |  |  | Implementation climate | (19, 21) |  |  |  |  |
|  |  |  |  |  |  |  | *Perceived organizational priority to implement* | (7) |  |  |  |  |
|  |  |  |  |  |  |  | How likely they were to implement such an app in future | (20) |  |  |  |  |
|  |  |  |  |  |  | Other | Individual identification with organization | (6) |  |  |  |  |
|  |  |  |  |  | Knowledge and perceptions | Perceived value and use of substance use services and perceived value of HIV-STI and substance use prevention | | (16) |  |  |  |  |
|  |  |  |  |  |  | Knowledge on newborn and child health care | | (4) |  |  |  |  |
|  |  |  |  |  |  | Current opinion about physical behavior during hospital stay | | (11) |  |  |  |  |
|  |  |  |  |  |  | Attitudes towards their workplace (support, functioning) | | (16) |  |  |  |  |
|  |  |  |  |  |  | *Value of ongoing monitoring of symptoms and functional status as part of usual care* | | (22) |  |  |  |  |
|  |  |  |  |  |  | *Underlying mental structures, and resulting practices and perceptions of systemic leverage points and barriers* | | (14) |  |  |  |  |
|  |  |  |  |  |  | *Value of educational tools* | | (13) |  |  |  |  |
|  |  |  |  |  |  | Attitudes about technology | | (6) |  |  |  |  |

*Qualitatively assessed; quantitatively assessed*

| **CONTEXT** | | | | | | | | | | | | | | |
| --- | --- | --- | --- | --- | --- | --- | --- | --- | --- | --- | --- | --- | --- | --- |
| **Domain** | **Level** | | | | | | | | | | | | | |
|  | **Micro-level** | | | | | **Meso-level** | | | | | **Macro-level** | | | |
|  | **Factor according CICI** | **Category defined** | | **Factor identified** | **Ref** | **Factor according CICI** | **Category defined** | | **Factor identified** | **Ref** | **Factor according CICI** | **Category defined** | **Factor identified** | **Ref** |
| **Socio-cultural** |  | | | | | Knowledge and perceptions | *Importance and interest in the upcoming SAMs, factors thought to affect adoption of the SMAs, and anticipated patient response to the SMAs* | | | (12) |  | | | |
|  |  |  |  |  |  |  | *Opinion on the most feasible HIV-testing intervention for primary care*  Organizational providers` attitudes towards the intervention and features of the intervention itself that might facilitate or compromise implementation  Awareness, perceptions and knowledge of the (5 or more ED visits/year) FUED problematic as well as specific needs and interests regarding case management | | | (10)  (6)  (19) |  |  |  |  |
| **Socio-economic** | - | | | | | Occupational aspects | Informal payment  Working conditions | | | (4)  (4) | Living conditions | Healthy-nutrition promoting environment | | (2) |
|  |  |  |  |  |  |  |  |  |  |  |  | Physical activity friendliness of the neighborhood | | (2) |
| **Political** | Health care system | Service delivery | Use of maternal and perinatal health services  Care seeking and treatment for child`s illness | | (4)  (4) | Health care system | Leadership | Implementation leadership  Leadership and roles  Leadership | | (21)  (9)  (4) | Public policies | Policy and environment | | (9) |
|  |  | Integration of patient`s needs and perspective | *The role of patients accessing appropriate educational materials*  *Information needs*  *User experience, and preferences for app features*  *What types of information should be shared about those symptoms (i.e., indicators, scales, and contextual information)*  *Whether a eHealth tool might meet patients` needs (example eHealth tool was presented)*  Self-behaviors that should be targeted  What outcomes are important for people with heart failure  They were read a list of statements pertaining to the communication of lunchbox messages to parents and to what extent they agreed with the statements | | (22)  (8)  (14)  (22)  (22)  (8)  (8)  (20) |  | Integration of patient`s needs and perspective | Teacher and parental involvement in PA promotion at school | | (2) | Health care system | Integration of patient`s needs and perspective | *Experiences with the health care system: things that are important as a receiver of care* | (22) |
|  |  |  |  |  |  |  |  | Teacher and parental involvement in nutrition at school | | (2) |  |  | *Opinions from stakeholders in need for an intervention* | (3) |
|  |  |  |  |  |  |  |  | Suggested content and delivery formats | | (8) |  |  | *Experiences with the health care system: what can be done to improve things* | (22) |
|  |  |  |  |  |  |  |  | *Opinions from stakeholders in need for an intervention* | | (3) |  | Service delivery | ED, hospital, and community treatment programs  Maternal, newborn and child health programs | (5)  (4) |
|  |  |  |  |  |  |  | Service delivery | Services provided to newborns and children | | (4) |  |  |  |  |
|  |  |  |  |  |  |  |  | Services their agency provides | | (16) | Relevant changes over time | Policies | *Impact of external policies on organization* | (7) |
|  |  |  |  |  |  |  |  | Current service provision | | (8) |  |  |  |  |
|  |  |  |  |  |  |  |  | *Strengths and weaknesses of existing heart failure services* | | (8) |  |  |  |  |

*Qualitatively assessed; quantitatively assessed*

| **CONTEXT** | | | | | | | | | | | | |
| --- | --- | --- | --- | --- | --- | --- | --- | --- | --- | --- | --- | --- |
| **Domain** | **Level** | | | | | | | | | | | |
|  | **Micro-level** | | | | **Meso-level** | | | | **Macro-level** | | | |
|  | **Factor according CICI** | **Category defined** | **Factor identified** | **Ref** | **Factor according CICI** | **Category defined** | **Factor identified** | **Ref** | **Factor according CICI** | **Category defined** | **Factor identified** | **Ref** |
| **Legal** | - | | | | - | | | | - | | | |
| **Ethical** | - | | | | Ethical principles and code of conduct | *Responsibility of supermarket and other food retrial actors in promoting a healthier lifestyle* | | (14) | - | | | |
|  |  |  |  |  |  | *Competitive pressure to implement screening* | | (7) |  |  |  |  |
|  |  |  |  |  | Conflicting interests | *Competitive pressure to implement screening* | | (7) |  |  |  |  |
| **Not specified** | Facilitators and barriers | *Facilitators and barriers to smoking cessation* | | (23) | Context | Inner context | *Documentation of inner context*  *Local contextual determinant* | (16)  (20) | Context | Outer context | Environmental level characteristics  *Documentation of outer context* | (6)  (16) |
|  |  | *Benefits of and barriers to exercise/physical activity* | | (8) |  |  |  |  |  |  |  |  |
|  |  | *Challenges and barriers patients experience using eHealth* | | (22) | Facilitators and barriers | *Perceived demand- and supply-side barriers to CBNC and iCCM service utilization*  Community reentry facilitators that might facilitate or compromise implementation | | (4)  (6) | Facilitators and barriers | *Perceived demand- and supply-side barriers to CBNC and iCCM service utilization* | | (4) |
|  |  | *Perceived demand- and supply-side barriers to CBNC and iCCM service utilization* | | (4) |  |  |  |  |  |  |  |  |

*Qualitatively assessed; quantitatively assessed*

*Qualitatively assessed; quantitatively assessed*

| **SETTING** | | | | | | | | | | |
| --- | --- | --- | --- | --- | --- | --- | --- | --- | --- | --- |
| **Aspect** | **Level** | | | | | | | | | |
|  | **Micro-level** | | | **Meso-level** | | | | **Macro-level** | | |
|  | **Category defined** | **Factor identified** | **Ref** | **Category defined** | **Factor identified** | **Ref** | **Category defined** | | **Factor identified** | **Ref** |
| **Work environment** | Workflows / work processes | *Insight in daily tasks* | (11) | Workflows / work processes | Fit with current practices | (17) | Health care delivery | | *Strengths and limitations of local practices corresponding to each step in the cascade* | (16) |
|  |  | *Efforts on health school pillars of education, familiarity to efforts* | (9) |  | *How can health providers (or persons who use health care) use technology to collect this information* | (22) |  |  |  |  |
|  |  |  |  |  | *How different communication methods would fit into provider workflows* | (22) |  |  |  |  |
|  |  |  |  | Accessibility of resources | *Accessibility of the assessment process* | (13) |  |  |  |  |
|  |  |  |  | Availability of resources | *Available and potential resources* | (9) |  |  |  |  |
|  |  |  |  |  | Available resources | (4) |  |  |  |  |
|  |  |  |  |  | *School data (existing resources for substance use programming, interventionist`s salary, required facilities, equipment, supplies)* | (21) |  |  |  |  |
|  |  |  |  | Training | Training needs assessment of health care professionals (current stage of knowledge/expertise with regard to elements of the intervention) | (8) |  |  |  |  |
|  |  |  |  |  | Training activities | (4) |  |  |  |  |
|  |  |  |  | Personal attributes | Personal attributes | (6) |  |  |  |  |
|  |  |  |  | Capacity | Relevant capacities of potential providers | (8) |  |  |  |  |
|  |  |  |  | Intervention fit | *Special considerations for delivering the innovation in the local context* | (3) |  |  |  |  |
|  |  |  |  |  | *Adaptability of lynch syndrome screening to local context* | (7) |  |  |  |  |
|  |  |  |  | Perceptions regarding intervention | What would influence their decision to implement the App | (20) |  |  |  |  |
|  |  |  |  |  | Principals who had previously but were not currently using a school-based mobile communication application were asked to report the reason that they were not | (20) |  |  |  |  |
|  |  |  |  |  | *Principals who were currently using a school-based mobile communication application were asked how often their school uploads information to the app* | (11) |  |  |  |  |
|  |  |  |  |  | *Practicability of indicator conditions-based HIV-testing* | (10) |  |  |  |  |
|  |  |  |  |  | Acceptability of intervention | (17) |  |  |  |  |
|  |  |  |  |  | Self-efficacy regarding ability to deliver the intervention | (6) |  |  |  |  |
|  |  |  |  |  | *Satisfaction with the implemented assessment process* | (13) |  |  |  |  |
|  |  |  |  |  | Ease of use | (17) |  |  |  |  |
|  |  |  |  |  | *Perceived difficulty implementing lynch syndrome screening* | (7) |  |  |  |  |
|  |  |  |  |  | *Teams inquiring about the feasibility of implementing the intervention in all identified locations* | (6) |  |  |  |  |
| Physical characteristics | Study site | Location of the household | (4) | Study site | Site characteristics | (16) | Prevention activities | | Nutrition promoting activities (incl. national) | (2) |
|  |  | Characteristics of the house and asset | (4) |  | Structural characteristics | (6) |  |  |  |  |
|  |  |  |  |  | School characteristics | (20) |  |  |  |  |
|  |  |  |  |  | Site assessment | (5) |  |  |  |  |
|  |  |  |  |  | Socio-demographic background GPs (age, years of experience, type of practice, number of patients/week, number of patients in follow-up, HIV tests/month, last HIV test performed) | (10) |  |  |  |  |
|  |  |  |  | Physical environment | Physical environment | (2) |  |  |  |  |
|  |  |  |  |  | Physical activity friendliness of school yard | (2) |  |  |  |  |
|  |  |  |  |  | School activities in the social and physical environment | (2) |  |  |  |  |
| Practice patterns | - | | | Preventive measures | *Mobilization during hospital stay of patients and caregivers* | (11) | - | | | |
|  |  |  |  |  | Afterschool PA | (2) |  |  |  |  |
|  |  |  |  |  | PA school activities | (2) |  |  |  |  |
|  |  |  |  | Behavioral interventions | Physical activity during recess | (2) |  |  |  |  |
|  |  |  |  |  | Whether they thought the nutritional quality of student lunchboxes needed improving at their school | (20) |  |  |  |  |
| Practice patterns | - | | | Communication skills | *Observation of conversation (admission, preparatory, bi-annual)* | (24) |  | | | |
|  |  |  |  |  | *Input on specific communication and counseling skills* | (10) |  | | | |
|  |  |  |  |  | Implementation of conversations upon admission and topics discussed in these | (24) |  | | | |
|  |  |  |  |  | *Interview with patient and relative how they perceived the conversation* | (24) |  |  |  |  |
|  |  |  |  | Skills in general | *GP`s awareness of patient`s sexual orientation and behavior to conduct an adequate HIV-risk assessment* | (10) |  |  |  |  |
|  |  |  |  |  | Relevant competencies of potential providers | (8) |  |  |  |  |
|  |  |  |  | Quality of care | *Patient`s and caregiver`s perceptions of care within the serial or multidisciplinary care model* | (25) |  |  |  |  |
|  |  |  |  | Health care delivery | *GP`s current approaches to HIV testing among MSM and SAM* | (10) |  |  |  |  |
|  |  |  |  |  | *Other aspects of primary health care delivery that may be important to capture for managing patients with complex chronic diseases and disability* | (22) |  |  |  |  |
|  |  |  |  |  | HIV testing practice | (10) |  |  |  |  |
|  |  |  |  |  | Practice CCM consistent care | (12) |  |  |  |  |
|  |  |  |  |  | HIV care | (10) |  |  |  |  |
|  |  |  |  |  | *Current gaps in care of COPD patients and departures from best practice* | (3) |  |  |  |  |
|  |  |  |  |  | *Patients journey during hospitalization* | (11) |  |  |  |  |
|  |  |  |  | Use of technology | Previously used or currently use of school-based mobile communication application to communicate with parents | (20) |  |  |  |  |
|  |  |  |  | Education | Physical education | (2) |  |  |  |  |
|  |  |  |  |  | Nutrition education | (2) |  |  |  |  |
|  |  |  |  | Information and advice | Whether they currently provided advice or information to parents on suitable foods to pack in lunchboxes, inclusion of lunchbox information in kindergarden orientation sessions and/or information packages and current and future use of lunchbox guidelines in their school | (20) |  |  |  |  |
|  |  |  |  | Care planning | Prevalence and content of advance care planning | (24) |  |  |  |  |
|  |  |  |  | Documentation | How often advance care sessions were documented | (24) |  |  |  |  |
|  |  |  |  | Guidelines | Availability of written guidelines | (24) |  |  |  |  |
|  |  |  |  | Decision making | *Which indicator conditions are regularly diagnosed and which of the are formed an indication for HIV testing* | (10) |  |  |  |  |
|  |  |  |  | Preparedness for health care delivery | Facility-level preparedness to provide child health services | (4) |  |  |  |  |

*Qualitatively assessed; quantitatively assessed*

**References**

1. Pfadenhauer LM, Gerhardus A, Mozygemba K, Lysdahl KB, Booth A, Hofmann B, Wahlster P, Polus S, Burns J, Brereton L *et al*: **Making sense of complexity in context and implementation: the Context and Implementation of Complex Interventions (CICI) framework**. *Implement Sci* 2017, **12**(1):21.

2. Verjans-Janssen S, Van Kann DH, Gerards SM, Vos SB, Jansen MW, Kremers SP: **Study protocol of the quasi-experimental evaluation of “KEIGAAF”: a context-based physical activity and nutrition intervention for primary school children**. *BMC Public Health* 2018, **18**(1):1-12.

3. Rotter T, Plishka C, Hansia MR, Goodridge D, Penz E, Kinsman L, Lawal A, O’Quinn S, Buchan N, Comfort P: **The development, implementation and evaluation of clinical pathways for chronic obstructive pulmonary disease (COPD) in Saskatchewan: protocol for an interrupted times series evaluation**. *BMC Health Serv Res* 2017, **17**(1):1-7.

4. Berhanu D, Okwaraji YB, Belayneh AB, Lemango ET, Agonafer N, Birhanu BG, Abera K, Betemariam W, Medhanyie AA, Abera M: **Protocol for the evaluation of a complex intervention aiming at increased utilisation of primary child health services in Ethiopia: a before and after study in intervention and comparison areas**. *BMC Health Serv Res* 2020, **20**(1):1-12.

5. D’Onofrio G, Edelman EJ, Hawk KF, Pantalon MV, Chawarski MC, Owens PH, Martel SH, VanVeldhuisen P, Oden N, Murphy SM: **Implementation facilitation to promote emergency department-initiated buprenorphine for opioid use disorder: protocol for a hybrid type III effectiveness-implementation study (Project ED HEALTH)**. *Implement Sci* 2019, **14**(1):48.

6. Johnson K, Gilbert L, Hunt T, Wu E, Metsch L, Goddard-Eckrich D, Richards S, Tibbetts R, Rowe JC, Wainberg ML: **The effectiveness of a group-based computerized HIV/STI prevention intervention for black women who use drugs in the criminal justice system: study protocol for E-WORTH (Empowering African-American Women on the Road to Health), a Hybrid Type 1 randomized controlled trial**. *Trials* 2018, **19**(1):1-19.

7. Rahm AK, Cragun D, Hunter JE, Epstein MM, Lowery J, Lu CY, Pawloski PA, Sharaf RN, Liang S-Y, Burnett-Hartman AN *et al*: **Implementing universal Lynch syndrome screening (IMPULSS): protocol for a multi-site study to identify strategies to implement, adapt, and sustain genomic medicine programs in different organizational contexts**. *BMC Health Serv Res* 2018, **18**(1):824.

8. Taylor RS, Hayward C, Eyre V, Austin J, Davies R, Doherty P, Jolly K, Wingham J, Van Lingen R, Abraham C: **Clinical effectiveness and cost-effectiveness of the Rehabilitation Enablement in Chronic Heart Failure (REACH-HF) facilitated self-care rehabilitation intervention in heart failure patients and caregivers: rationale and protocol for a multicentre randomised controlled trial**. *BMJ Open* 2015, **5**(12):e009994.

9. Van Dongen BM, Ridder MAM, Steenhuis IHM, Renders CM: **Background and evaluation design of a community-based health-promoting school intervention: Fit Lifestyle at School and at Home (FLASH)**. *BMC Public Health* 2019, **19**(1):1-11.

10. Apers H, Vuylsteke B, Loos J, Smekens T, Deblonde J, Van Beckhoven D, Nöstlinger C: **Development and evaluation of an HIV-testing intervention for primary care: protocol for a mixed methods study**. *JMIR Res Protoc* 2020, **9**(8):e16486.

11. Van Delft LMM, Bor P, Valkenet K, Veenhof C: **Hospital in Motion, a multidimensional implementation project to improve patients’ physical behavior during hospitalization: protocol for a mixed-methods study**. *JMIR Res Protoc* 2019, **8**(4):e11341.

12. Kwan BM, Dickinson LM, Glasgow RE, Sajatovic M, Gritz M, Holtrop JS, Nease DE, Ritchie N, Nederveld A, Gurfinkel D: **The Invested in Diabetes Study Protocol: a cluster randomized pragmatic trial comparing standardized and patient-driven diabetes shared medical appointments**. *Trials* 2020, **21**(1):1-14.

13. Shanley DC, Hawkins E, Page M, Shelton D, Liu W, Webster H, Moritz KM, Barry L, Ziviani J, Morrissey S *et al*: **Protocol for the Yapatjarrathati project: a mixed-method implementation trial of a tiered assessment process for identifying fetal alcohol spectrum disorders in a remote Australian community**. *BMC Health Serv Res* 2019, **19**(1):649.

14. Lakerveld J, Mackenbach JD, De Boer F, Brandhorst B, Broerse JE, De Bruijn G-J, Feunekes G, Gillebaart M, Harbers M, Hoenink J: **Improving cardiometabolic health through nudging dietary behaviours and physical activity in low SES adults: design of the Supreme Nudge project**. *BMC Public Health* 2018, **18**(1):1-9.

15. Nahar P, van Marwijk H, Gibson L, Musinguzi G, Anthierens S, Ford E, Bremner SA, Bowyer M, Le Reste JY, Sodi T: **A protocol paper: community engagement interventions for cardiovascular disease prevention in socially disadvantaged populations in the UK: an implementation research study**. *Glob Health Res Policy* 2020, **5**(1):1-9.

16. Knight DK, Belenko S, Wiley T, Robertson AA, Arrigona N, Dennis M, Bartkowski JP, McReynolds LS, Becan JE, Knudsen HK: **Juvenile Justice—Translational Research on Interventions for Adolescents in the Legal System (JJ-TRIALS): a cluster randomized trial targeting system-wide improvement in substance use services**. *Implement Sci* 2015, **11**(1):1-18.

17. Osilla KC, Becker K, Ecola L, Hurley B, Manuel JK, Ober A, Paddock SM, Watkins KE: **Study design to evaluate a group-based therapy for support persons of adults on buprenorphine/naloxone**. *Addict Sci Clin Pract* 2020, **15**(1):1-11.

18. Bidwell P, Thakar R, Sevdalis N, Silverton L, Novis V, Hellyer A, Kelsey M, van der Meulen J, Gurol-Urganci I: **A multi-centre quality improvement project to reduce the incidence of obstetric anal sphincter injury (OASI): study protocol**. *BMC Pregnancy Childbirth* 2018, **18**(1):1-11.

19. Grazioli VS, Moullin JC, Kasztura M, Canepa-Allen M, Hugli O, Griffin J, Vu F, Hudon C, Jackson Y, Wolff H *et al*: **Implementing a case management intervention for frequent users of the emergency department (I-CaM): an effectiveness-implementation hybrid trial study protocol**. *BMC Health Serv Res* 2019, **19**(1):28.

20. Sutherland R, Brown A, Nathan N, Janssen L, Reynolds R, Walton A, Hudson N, Chooi A, Yoong S, Wiggers J: **Protocol for an effectiveness-implementation hybrid trial to assess the effectiveness and cost-effectiveness of an m-health intervention to decrease the consumption of discretionary foods packed in school lunchboxes: The ‘SWAP IT’trial**. *BMC Public Health* 2019, **19**(1):1-11.

21. Hartzler B, Lyon AR, Walker DD, Matthews L, King KM, McCollister KE: **Implementing the teen marijuana check-up in schools—a study protocol**. *Implement Sci* 2017, **12**(1):1-14.

22. Gray CS, Wodchis WP, Upshur R, Cott C, McKinstry B, Mercer S, Palen TE, Ramsay T, Thavorn K: **Supporting goal-oriented primary health care for seniors with complex care needs using mobile technology: evaluation and implementation of the health system performance research network, Bridgepoint electronic patient reported outcome tool**. *JMIR Res Protoc* 2016, **5**(2):e126.

23. Quintiliani LM, Russinova ZL, Bloch PP, Truong V, Xuan Z, Pbert L, Lasser KE: **Patient navigation and financial incentives to promote smoking cessation in an underserved primary care population: A randomized controlled trial protocol**. *Contemp Clin Trials* 2015, **45**:449-457.

24. Sævareid TJL, Lillemoen L, Thoresen L, Førde R, Gjerberg E, Pedersen R: **Implementing advance care planning in nursing homes–study protocol of a cluster-randomized clinical trial**. *BMC Geriatr* 2018, **18**(1):1-12.

25. Smeltzer MP, Rugless FE, Jackson BM, Berryman CL, Faris NR, Ray MA, Meadows M, Patel AA, Roark KS, Kedia SK: **Pragmatic trial of a multidisciplinary lung cancer care model in a community healthcare setting: study design, implementation evaluation, and baseline clinical results**. *Transl Lung Canc Res* 2018, **7**(1):88.
